# Efficient hepatic differentiation and regeneration potential under xeno-free conditions using mass-producible amnion-derived mesenchymal stem cells

**DOI:** 10.1186/s13287-021-02470-y

**Published:** 2021-11-12

**Authors:** Jiwan Choi, Seoon Kang, Bitnara Kim, Seongjun So, Jongsuk Han, Gyeong-Nam Kim, Mi-Young Lee, Seonae Roh, Ji-Yoon Lee, Soo Jin Oh, Young Hoon Sung, Yeonmi Lee, Sung Hoon Kim, Eunju Kang

**Affiliations:** 1 Department of Medical Science, Asan Medical Institute of Convergence Science and Technology (AMIST), University of Ulsan College of Medicine, Asan Medical Center, Seoul, 05505 South Korea; 2 Stem Cell Center, Asan Institute for Life Sciences, Asan Medical Center, Seoul, 05505 South Korea; 3 Present Address: Center for Embryo & Stem Cell Research, CHA Advanced Research Institute and Department of Biomedical Science, CHA University, Pocheon-si, Gyeonggi 13488 South Korea; 4 Convergence Medicine Research Center, Asan Institute for Life Sciences, Asan Medical Center, Seoul, 05505 South Korea; 5 Department of Obstetrics and Gynecology, University of Ulsan College of Medicine, Asan Medical Center, Seoul, 05505 South Korea; 6 Asan Institute for Life Sciences, Asan Medical Center and Department of Convergence Medicine, College of Medicine, University of Ulsan, Seoul, 05505 South Korea

**Keywords:** Amnion-derived mesenchymal stem cells, Differentiation, Hepatic progenitor, Xeno-free, GSK3 inhibitor, Polyvinyl alcohol

## Abstract

**Background:**

Amnion-derived mesenchymal stem cells (AM-MSCs) are an attractive source of stem cell therapy for patients with irreversible liver disease. However, there are obstacles to their use due to low efficiency and xeno-contamination for hepatic differentiation.

**Methods:**

We established an efficient protocol for differentiating AM-MSCs into hepatic progenitor cells (HPCs) by analyzing transcriptome-sequencing data. Furthermore, to generate the xeno-free conditioned differentiation protocol, we replaced fetal bovine serum (FBS) with polyvinyl alcohol (PVA). We investigated the hepatocyte functions with the expression of mRNA and protein, secretion of albumin, and activity of CYP3A4. Finally, to test the transplantable potential of HPCs, we transferred AM-MSCs along with hepatic progenitors after differentiated days 11, 12, and 13 based on the expression of hepatocyte-related genes and mitochondrial function. Further, we established a mouse model of acute liver failure using a thioacetamide (TAA) and cyclophosphamide monohydrate (CTX) and transplanted AM-HPCs in the mouse model through splenic injection.

**Results:**

We analyzed gene expression from RNA sequencing data in AM-MSCs and detected downregulation of hepatic development-associated genes including *GATA6, KIT, AFP, c-MET, FGF2, EGF, and c-JUN,* and upregulation of *GSK3*. Based on this result, we established an efficient hepatic differentiation protocol using the GSK3 inhibitor, CHIR99021. Replacing FBS with PVA resulted in improved differentiation ability, such as upregulation of hepatic maturation markers. The differentiated hepatocyte-like cells (HLCs) not only synthesized and secreted albumin, but also metabolized drugs by the CYP3A4 enzyme. The best time for translation of AM-HPCs was 12 days from the start of differentiation. When the AM-HPCs were transplanted into the liver failure mouse model, they settled in the damaged livers and differentiated into hepatocytes.

**Conclusion:**

This study offers an efficient and xeno-free conditioned hepatic differentiation protocol and shows that AM-HPCs could be used as transplantable therapeutic materials. Thus, we suggest that AM-MSC-derived HPCs are promising cells for treating liver disease.

**Supplementary Information:**

The online version contains supplementary material available at 10.1186/s13287-021-02470-y.

## Background

The liver plays a key role in the detoxification of drugs, the synthesis of proteins and hormones, and supports glycogen and cholesterol metabolism. Liver failure can be the result of acute or chronic causes such as alcohol, toxic drugs, viral infection, and genetic factors [1]. Approximately 100,000 individuals per year are diagnosed with alcoholic liver disease and 12,000 people die only in the US [2].

Recently, advanced stem cell technologies have extended the resources of regenerative medicine, and stem cells are expected to replace liver transplantation in the foreseeable future [3]. To obtain transplant cells for liver disease, hepatocytes or hepatic progenitor cells [4], pluripotent stem cells (PSCs), such as induced pluripotent stem cells (iPSCs) [5] or embryonic stem cells (ESCs) [6], mesenchymal stem cells (MSCs) [7], hepatic progenitor cells isolated from the liver or derived from hepatocytes, or hepatocytes themselves, are used [8]. Until now, these sources have several limitations for clinical application. For example, iPSCs display a high risk of tumorigenicity and have low-efficiency differentiation ability, the use of ESCs is limited by their genetic background in terms of HLA types, MSCs have low differentiation ability, contamination with xeno-material such as fetal bovine serum (FBS) or Matrigel, and there is limited access to enough number of natural liver progenitor cells.

We have reported that hepatocyte-like cells (HLCs) differentiated from adult human liver-derived stem cells (hLD-SCs) have good liver regeneration potential [9]. However, their obtained cell numbers from health donor individuals are limited and they are exposed to undefined animal-derived products such as FBS during differentiation [10].

Human amniotic membranes (AM) are high-yielding sources of stem cells [11]. Since the placenta is a temporary organ in which maternal and fetal cells occur together, fetal cells must be protected from the maternal immune system. For this reason, the expression of the major histocompatibility complex (MHC) is reduced in the placental membrane [12]. Moreover, placental membrane-derived stem cells have high proliferation rates, and differentiation abilities comparable to stem cells from other sources [13]. Especially, MSC-like cells isolated from the AM have been characterized in the previous studies in terms of multipotency, low tumorigenicity, immunomodulation, and anti-inflammatory capacities [14]. Human AM-MSCs could be differentiated into typical mesenchymal lineages, such as osteocytes, chondrocytes, and adipocytes, and no tumorigenic conversion was observed [15]. Moreover, the immunomodulation and anti-inflammatory capacity of AM-MSCs had reported that cells display supportive function through paracrine effects [16].

FBS is a highly effective growth supplement for cell culture. However, there is batch-to-batch variability that may affect cell characteristics, and the risk of contamination with harmful pathogens in human transplantation studies [17, 18]. Accordingly, progressive attempts have been made to devise chemically defined xeno-free culture conditions for stem cells [19, 20].

In this study, we reexamined the characteristics of AM-MSCs compared with the umbilical cord matrix (UCM)-MSCs in terms of phenotype and gene expression. Moreover, we showed that AM-MSCs could become potentially useful donors by modifying the B2M gene. Next, after RNA expression analysis, we established an efficient protocol for differentiating AM-MSCs into HLCs by adding the GSK3 inhibitor, CHIR99021, and polyvinyl alcohol (PVA) instead of fetal bovine serum under gelatin-coated plates. The gelatin-coated condition can promote the proliferation and growth of MSCs [21, 22]. Finally, we identified the optimum time for transplantation after initiating in vitro differentiation by measuring the expression of the carboxypeptidase M *(CPM)* and epithelial cell adhesion molecule (*EpCAM)* genes and the oxygen consumption rate of mitochondria. Hepatic progenitor cells transplanted into a mouse model induced by thioacetamide (TAA) and cyclophosphamide monohydrate (CTX) settled in the damaged livers and differentiated into functional HLCs.

## Methods

### Cell isolation

Stem cells were isolated from the amniotic membrane after delivery. The use of fetal membrane was approved by the Institutional Review Board (IRB) of Asan Medical Center (Seoul, Korea; 2015–0303), and informed consent was obtained from participants, women aged 33-39 years. All samples were obtained after a normal gestation period (37-38 weeks).

Briefly, the membranes were transferred into a Petri dish with 0.1% collagenase IV and cut into small pieces under clean bench conditions using operating scissors. The small pieces of tissue were transferred to a MACS C tube (Miltenyi Biotec, Bergisch Gladbach, Germany) and mechanically dissociated with a MACS Dissociator (Miltenyi Biotec). Dissociated cells were centrifuged at 500 x g for 3 min. The supernatant was removed and erythrocytes lysed with a 1x RBC lysis buffer (eBioscience, San Diego, California, USA). Then, the culture medium was added and centrifuged at 800 x g for 3 min. The supernatant was removed, and the pellet resuspended with a culture medium. The cells were plated in 0.1% gelatin (Sigma Aldrich, MO, USA)-coated culture dishes. Only cell preparations with fibroblast-like morphology were used in further experiments.

### Culture for mesenchymal stem cells

Human umbilical cord matrix-derived stem cells (UCM-MSCs) were provided by the Asan Stem Cell Center (Asan Institute for Life Sciences, Seoul, Korea). The cells were obtained from the previously described protocols [23]. Both stem cell types (UCM-MSCs and AM-MSCs) were cultured on 0.1% gelatin-coated culture dishes with a culture medium. The culture medium was based on DMEM/F12 supplemented with 10% fetal bovine serum (FBS; Gibco, NY, USA), 10 ng/ml fibroblast growth factor 2 (FGF2; Peprotech, Rocky Hill, NJ, USA), 1% NEAA (Gibco), 1% Penicillin/Streptomycin (GeneDirex). Cells were passaged 1 to 3–5 every 3 to 4 days using trypsin/EDTA (Gibco, NY, USA).

### Culture for primary human hepatocytes

Primary human hepatocytes (PHH) were purchased from Thermofisher scientific (HMCPMS; Thermofisher Scientific, MA, USA), and were cultured and maintained according to the supplemented protocol. In brief, the hepatocytes were thawed in Cryopreserved hepatocytes recovery medium (CM7000; Thermofisher Scientific), centrifuged at 100 x g for 10 min, and seeded at 2 × 10^6^ cells/well on a collagen-coated plate in cryopreserved hepatocyte plating medium (CM9000; Thermofisher Scientific). After incubating the plate at 37 °C for 6 h, the culture medium was replaced by William’s medium (Gibco) supplemented with hepatocytes maintenance supplement pack (CM4000; Thermofisher Scientific).

### Quantitative RT-PCR

Total RNA was extracted using an RNeasy Mini Kit (Qiagen, CA, USA) following the manufacturer’s instructions. cDNA was synthesized with an Ultrascript 2.0 cDNA Synthesis Kit (PCR Biosystems, London, UK), and qRT–PCR was performed using a Power SYBR® Green PCR Master Mix (Applied Biosystems, CA, USA) on a QuantStudioTM real-time PCR System (Applied Biosystems). RNA levels were normalized with GAPDH. Primer sequences are listed in Table S1.

### Flow cytometric analysis

To measure cell membrane surface proteins, cells were collected after detachment with trypsin/EDTA, and immunostained for 30 min at 4 °C with fluorescence-conjugated primary antibodies. The following antibodies were used: PE-CD34, FITC-CD90, FITC-CD105, PE-MHC I, APC-MHC II (BD Biosciences Pharmingen, San Diego, CA, USA), and PE-HLA-G (MHC Ib) (Biolegend, CA, USA). After immunostaining, the cells were washed three times with PBS and suspended in 0.2 ml PBS for analysis. The fluorescence of samples was measured using a FACS Calibur (Becton Dickinson and Company, NJ, USA) and analyzed with FlowJo software (ver 10.6.1; Treestar, OR, USA).

### Osteogenesis and adipogenesis of AM-MSCs

Differentiation was performed using Osteogenesis and Adipogenesis differentiation Kit (Thermofisher Scientific) to generate the osteocytes and adipocytes in AM-MSCs. The differentiation protocols were performed according to the kit’s recommendations.

### Measuring the cell growth rate

To measure the extent of cell growth rate, stem cells were seeded at 1 × 10^5^ cells in 0.1% gelatin-coated 6-well plates at every passage. Cell numbers were counted by hemocytometer. The extent of cell number was calculated as a cell growth rate from the formula: Day 3 cell number/1 × 10^5^ cells.

### Production of B2M-KO-AM-MSCs

Human genomic *B2M* sequences were analyzed and selected using the web tool Benchling (https://benchling.com/). B2M-specific CRISPR-Cpf1 expression vector was constructed by cloning the annealed oligomers (5′-agatCCGATATTCCTCAGGTACTC-3′ and 5′-aaaaGAGTACCTGAGGAATATCGG-3′) into a pY108 lentiviral vector (Addgene, plasmid #84739). Infectious lentiviral particles were produced as described previously and were precipitated using Lenti Concentrator (Origene, Rockville, Maryland, US) according to the manufacturer’s protocol [24]. To produce stable *B2M*-KO-AM-MSCs, resuspended lentivirus in culture media were added to AM-MSCs and were incubated for 24 h in the culture medium. The cell culture medium was replaced with a fresh medium containing 4 μg/ml puromycin and incubation continued for a further 24 h. AM-MSCs in which B2M was knocked out and that did not express MHC I was selected with a BD FACSAria™ III Cell Sorter. B2M-KO was accessed by an Indel sequencing primer (Table S1).

### PBMC proliferation assay

The proliferation assay was performed in the previous report with simple modification [24]. Briefly, the MSCs (3 × 10^4^ cells/well) were plated onto a 96-well plate in 100 μl of MSC culture medium and were allowed to adhere to the plates overnight. The next day, the medium (RPMI 1640 medium supplemented with 10% FBS, 2 mM L-glutamine, 1% P/S) was fully changed, and human PBMCs (*n* = 4) were added to wells (1 × 10^5^ cells/well in 100 μl volume) in a containing or lacking MSCs with or without 5 μg/ml phytohemagglutinin (PHA, Sigma). Human PBMCs with or without PHA were used as positive or negative controls. After 7 days, 100 μl of cells from each well was transferred to new 96-well plates with a 10 μl CCK-8 assay kit (Dojindo). The absorbance at 450 nm was measured with a microplate reader.

### Transcriptome analysis

AM-MSCs (*n* = 3) and UCM-MSCs (*n* = 3) were sequenced by strand-specific, paired-end sequencing (Illumina, San Diego, CA, USA), generating approximately 1.1 to 1.3 × 10^6^ reads per sample. The quality of the raw data sets was analyzed with the software FastQC (v0.11.5), and adapter sequences of < 20 bp in length, and sequences with a quality score lower than Q20, were removed using Cutadapt software (v1.5) to increase mapping quality. After trimming, the remaining sequences, constituting 97.3 to 98.8% of the raw data sets, were aligned with the human reference genome (GRCh38.p13) using STAR software (v2.7.5c), and reads were summarized using featureCount software. Normalization of reads, analysis of gene expression, and calculation of differentially expressed genes was performed using DESeq2 (1.28.1). Gene Ontology Enrichment Analysis of the differentially expressed genes was conducted using online tools (http://geneontology.org). Functional enrichment in the Biological Process of the GO terms was analyzed. A list of endoderm-associated genes was retrieved from the online human gene database (https://www.genecards.org/).

### In vitro hepatic differentiation

Stem cells were differentiated by the previously published conventional hepatogenic differentiation protocol [9] and an advanced protocol. Briefly, in the advanced protocol, the cells were seeded on 0.1% gelatin-coated dishes at 7000 cells/cm^2^ in a cell culture medium. After 2 days, they were cultured for 7 days with Step-1 medium consisting of Iscove’s Modified Dulbecco’s Medium (IMDM; Gibco) supplemented with 0.1% polyvinyl alcohol (PVA; Sigma Aldrich) or 1% FBS, 10 mM nicotinamide (Sigma Aldrich), 20 ng/ml hHGF (Peprotech), 10 ng/ml FGF2, 2 μM 5-azacytidine (Sigma Aldrich), 0.1 μM dexamethasone (Sigma Aldrich), 1% insulin-transferrin-selenium (ITS; Gibco), 3 μM CHIR99021, 20 ng/ml EGF (Peprotech), and 10 μM Fasudil (AdooQ Bioscience, Irvine, CA, USA). For hepatic maturation, the Step-1 medium was replaced with Step-2 medium consisting of IMDM supplemented with 1 μM dexamethasone, 1% ITS, 20 ng/ml Oncostatin M (OSM, Peprotech), 20 ng/ml hHGF, and 10 uM Fasudil.

### Immunocytochemical staining

Cells were fixed with 4% formaldehyde overnight, washed with the PBST buffer, permeabilized in 0.5% Triton X-100 and blocked in PBST containing 1% bovine serum albumin (Sigma Aldrich). Samples were incubated with primary antibodies, anti-human Albumin, anti-human CYP3A4 (1:100; Santa Cruz Biotechnology, Dallas, TX, USA) and anti-human EpCAM (1:100; Abcam, Cambridge, MA, USA), overnight at 4 °C, followed by goat anti-mouse IgG Alexa Fluor 488-conjugated and donkey anti-rabbit IgG Alexa Fluor 647-conjugated secondary antibody (1:500; Abcam). Nuclei were counterstained with 4′, 6-diamidino-2-phenylindole (DAPI) (Sigma Aldrich) for 10 min, and fluorescence signals were detected using an AxioObserver Z1 microscope (Carl Zeiss, Oberkochen, Germany).

### Detection of secreted human albumin

The presence of human albumin was determined by the Human albumin ELISA Kit (Bethyl Laboratories, Texas, USA). The assay procedure was performed according to the supplier’s recommendation. The albumin secretion was normalized to culture day and total cell numbers.

### Measurement of CYP3A4 activity in vitro

To assess the hepatic enzyme activity, 25 μM rifampicin (Sigma), which is a CYP3A4 inducer, was treated for 48 h in cells. Next, 20 μM midazolam (Sigma), previously reported as the substrate of CYP3A4 [25], was treated for 24 h. Sample preparation involved simple protein precipitation with organic solvent (cold acetonitrile). Briefly, cell samples (50 uL) were precipitated with 150 μL of cold acetonitrile containing internal standard carbamazepine (CBZ, 10 ng/mL), and agitating with a vortex mixer before centrifugation. Samples were centrifuged at 3400 rpm, 4 °C for 20 min and the supernatant samples were analyzed by LC-MS/MS according to the previous publication [26].

Briefly, the LC-ESI/MS/MS system consisted of an Agilent 1200 series HPLC system (Agilent Technologies, Wilmington, DE, USA) coupled with an API 4000 LC-MS/MS with a Turbo V IonSpray source (Applied Biosystems, Foster City, CA, USA) operated in the positive ion mode. The chromatographic column was conducted on an Atlantis dC18 column (50 × 2.1 mm i.d., 3 μm, Waters, Milford, MA, USA) with a SecurityGuard C18 guard column (2.0 × 4.0 mm i.d., Phenomenex, Torrance, CA, USA). The sample injection volume was 5 μL, and the flow rate was set at 0.4 mL/min, and the oven temperature was maintained at 30 °C. The mobile phase consisted of HPLC water (A) and acetonitrile (B), each containing 0.1% formic acid. The TurboIonSpray interface was operated in the positive ion mode at 5500 V. The operating conditions were determined as follows: ion source temperature, 600 °C; nebulizing gas flow, 50 L/min; auxiliary gas flow, 5.0 L/min; curtain gas flow, 20 L/min; and collision gas (nitrogen) pressure 3.4 × 10^− 5^ Torr. Nitrogen gas was used for the curtain gas (CUR), collision gas (CAD), and nebulizer gas (NEB). The detection was conducted using multiple reaction monitoring (MRM) of the transitions of m/z 342 > 324 for 1-hydroxymidazolam and m/z 237 > 194 for carbamazepine (internal standard). Acquisition and analysis of data were performed using Analyst software (ver. 1.5.2, Applied Biosystems, Foster City, CA, USA).

The enzyme activity was also determined using the P450-Glo CYP3A4 kit (Promega, USA). The experiment was performed according to the supplier’s recommendations. The luminescence was measured by SpectraMax® L Luminometer (Molecular Devices, CA, USA). The activity was normalized to the culture day and dsDNA content of each sample.

### Seahorse assay

To measure oxygen consumption rates (OCR) in differentiated cells, stem cells were seeded at 7000 cells/cm^2^ and differentiated into hepatocyte-like cells (HLCs) to day 0, 7, 10, 12, and 14 in 0.1% gelatin-coated XFe24 cell culture plates (Agilent Technologies, Santa Clara, CA, USA). Mitochondrial OCR was measured with an XF Cell MitoStress test kit in an XF24 extracellular flux analyzer (Agilent Technologies) and calculated as described [9]. Values were normalized by the amount of cellular DNA.

### Transplantation experiments

To establish an acute liver failure model, eight-week-old male C57BL/6 or NOD. Cg-Prkdc^scid^ Il2rg^tm1Wjl^/SzJ (NSG) mice were injected intraperitoneally with 0.08 mg/g thioacetamide (TAA; 163,678, Sigma Aldrich) or an additional 0.025 mg/g cyclophosphamide monohydrate (CTX; C0768, Sigma Aldrich) after 24 h. Next, the cells with various stages were transplanted via the intrasplenic route (5 × 10^5^ cells per mouse) of NSG. All mice were purchased from JOONG AH BIO (Suwon, Korea). The mouse study was approved by the Asan Institutional Animal Care and Use Committee (IACUC, 2018–12-167, 2019–12-062, and 2020–02-208).

### Blood analysis

Complete blood counts (CBC) and analyses of peripheral blood from mice were performed using an ABC-VET (Scilvet, Germany) and FUJI DRI-CHEM clinical chemistry analyzer (FUJI, Japan), respectively, at AniCom Medical Center (Seoul, Korea).

### Histological staining and analysis

The retrieved liver tissues were fixed in 3.7% formaldehyde and embedded in paraffin. The paraffin blocks were sectioned at 4 μm-thick. To assess acute liver failure in chemically treated mice, the paraffin sections were stained with hematoxylin and eosin (H&E) and scored by Suzuki’s method [27]. Three weeks after transplantation, liver repopulation and regeneration were assessed by H&E staining, and immunohistochemistry (IHC). Human albumin was stained with DAB substrate using the previously described IHC methods [9]. Histological images were obtained by light microscopy using an Olympus DP27 (Olympus, Melville, NY, USA).

### Immunofluorescence staining

Transplanted liver tissues were fixed in 3.7% formaldehyde overnight at 4 °C, incubated overnight at 4 °C in 30% sucrose, overlaid with OCT compound, and frozen in liquid nitrogen. The frozen blocks were sectioned with a cryotome of 10 μm thickness. The sections were permeabilized with 0.2% Triton X-100/PBS for 30 min at RT, blocked with 10% FBS buffer at RT in a humidity chamber, and stained overnight with anti-human albumin diluted 1:100 at 4 °C in the humidity chamber. The secondary antibody was goat anti-mouse IgG H&L Alexa Fluor® 647; Abcam, 1:100). Nuclei were counterstained with 4′, 6-diamidino-2-phenylindole (DAPI) for 10 min, and fluorescence signals were detected using an AxioObserver Z1 microscope.

### Human mitochondrial DNA detection

Total DNA was isolated with a PicoPure™ DNA extraction kit (Thermofisher Scientific). The PCR reaction was conducted using 2x PCRBIO HS Taq Mix Red (PCRbiosystems) with 300 ng of DNA in a total volume of 20 μl. The reaction was performed as described in the kit with annealing at 56 °C for 20 s. Sanger sequencing of the PCR products of injected cells and transplanted cells was carried out by Macrogen (Seoul, Korea).

### Evaluation of transplant efficiency

To determine the percentage of engrafted human cells in the mouse liver, each standard curve of human or mouse mtDNA was prepared by quantitative real-time PCR (qPCR). For preparing the standard curve, both DNA concentrations were measured by NanoDrop™ 2000/2000c Spectrophotometer (Thermofisher Scientific), and samples with OD 260/280 between 1.8–2.0 were used for further analysis. The DNA concentration was diluted by DNase-free water to the following concentration, 1000, 100, 10, and 1 ng/ul. The qPCR was performed using 1 ul of the diluted DNA (1000, 100, 10, and 1 ng) and Power SYBR® Green PCR Master Mix on a QuantStudio™ real-time PCR System. After finishing the qPCR, the standard curve of human or mouse mtDNA was made using the diluted DNA concentration (X-axis) and CT value (Y-axis) (Fig. S3e). Finally, human and mouse mtDNA concentrations were acquired through the standard curve in transplanted samples, and the percentage of transplantation efficiency was calculated following formula: [human mtDNA / (mouse mtDNA + human mtDNA)] × 100 (%).

### Statistical analysis

All experiments were performed on at least three (*n* = 3) independent biological samples, and data are presented as means ± standard deviations (SD). Statistical analysis was performed using GraphPad Prism 6.0 software (GraphPad Software, CA, USA). Comparisons of three or more data sets were performed by one-way or two-way ANOVA followed by Bonferroni’s multiple comparison tests. Two-group comparisons were made using two-tailed Student’s t-tests. *P* < 0.05 was considered statistically significant.

## Results

### Characterization of amniotic membrane-derived mesenchymal stem cells

**Fig. 1:**
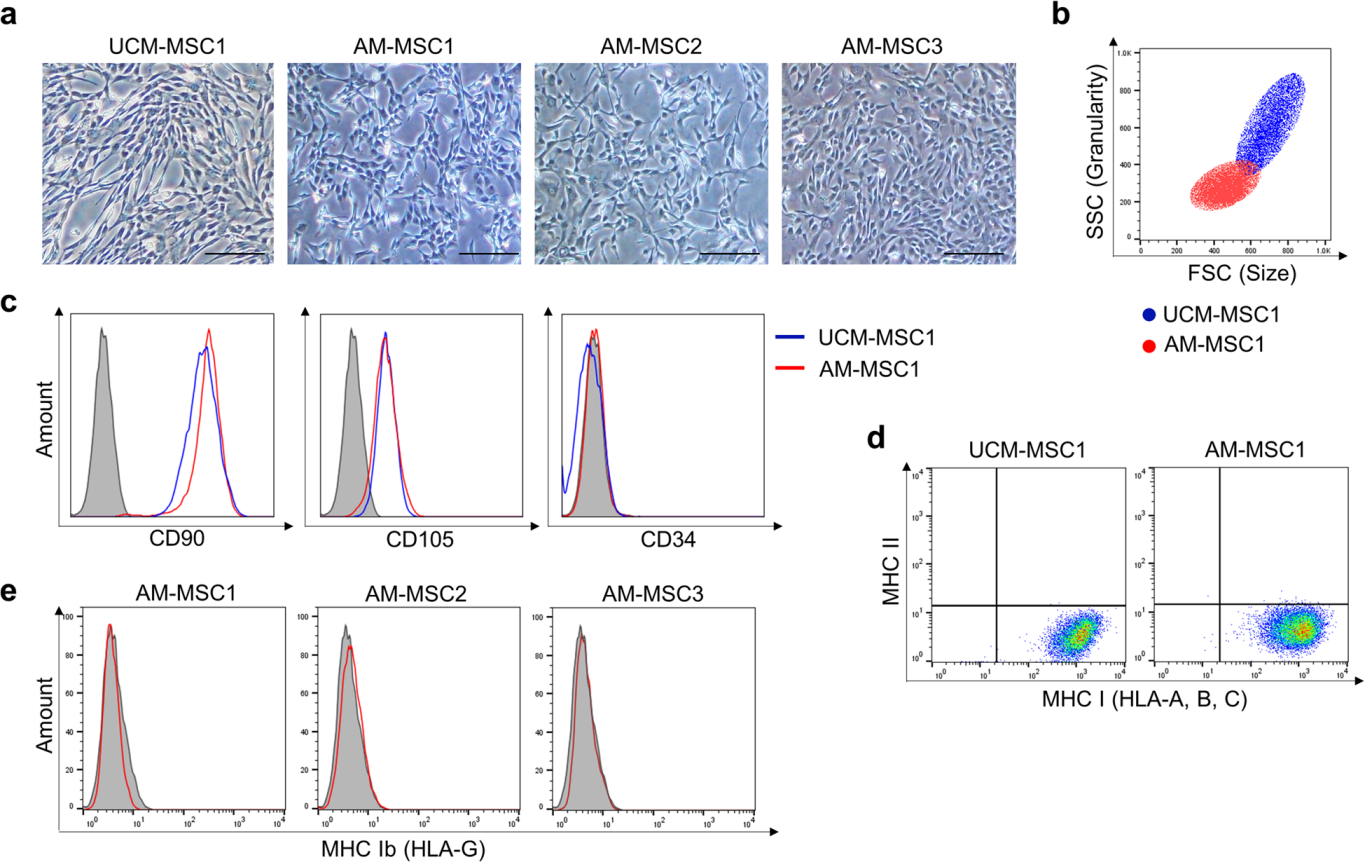
Phenotypes of AM-MSCs. **a** Morphology of stem cells isolated from the amniotic membrane and umbilical cord matrix. Scale bar = 200 μm. **b** The FSC-SSC dot plot of AM-MSCs, and UCM-MSCs. FSC represents cell size, and SSC represents the granularity of cells in flow cytometry (Red dot: AM-MSC1, Blue dot: UCM-MSCs). **c** Flow cytometry analysis shows that AM-MSC1 and UCM-MSC1 had similar characteristics in terms of expression of MSC surface markers (CD90 and CD105) and absence of a hematopoietic stem cell marker (CD34). **d** Profile of MHC class I (HLA-A, B, C) and II in AM-MSC1 and UCM-MSC1. **e** Profile of non-canonical MHC class Ib (HLA-G) in three AM-MSCs cell lines

Three amniotic membrane-derived mesenchymal stem cells (AM-MSCs) were compared with umbilical cord matrix-derived mesenchymal stem cells (UCM-MSCs). Morphologically, AM-MSCs were fibroblast-like shapes with ovoid nuclei, which is similar to UCM-MSCs (Fig. 1a). However, the AM-MSCs were relatively smaller in size and granularity than UCM-MSCs by measuring flowcytometry [28] (Fig. 1b). Like UCM-MSCs, AM-MSCs expressed the mesenchymal stem cell (MSC) surface markers, CD90, and CD105, but did not express CD34 (Fig. 1c). The major histocompatibility complex (MHC) profile of the AM-MSCs was similar to that of the UCM-MSCs; positive for MHC I and negative for MHC II (Fig. 1d). To eliminate contamination of the amnion derived-epithelial cells, which could be expressed in non-canonical HLA types (MHC Ib), such as HLA-G [29], we observed HLA-G expression in AM-MSCs resulting in negative in all three AM-MSC lines (Fig. 1e).

**Fig. 2:**
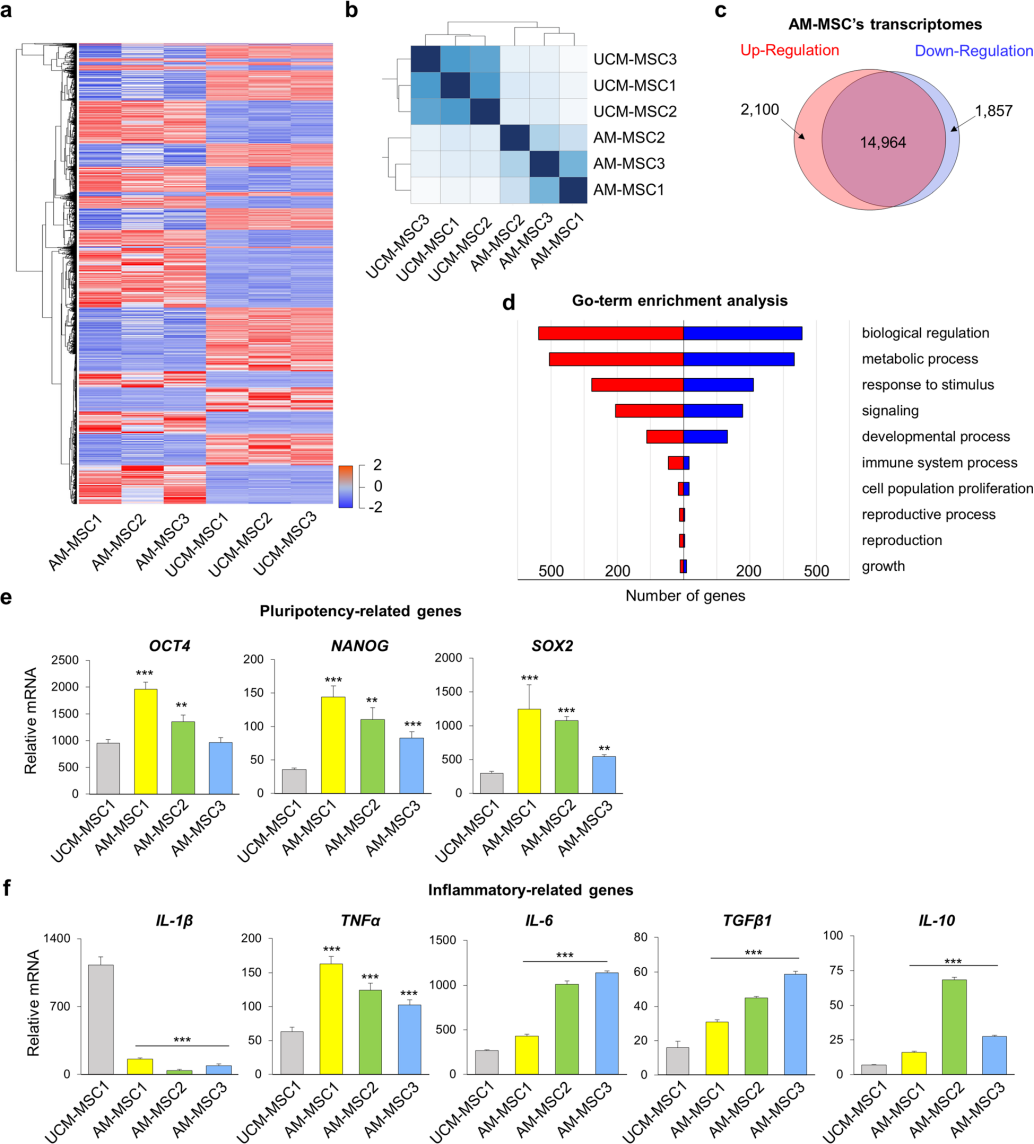
AM-MSCs and UCM-MSCs have different transcriptome profiles. **a** Hierarchical cluster analysis, and heatmap of differentially expressed genes (DEGs) between AM-MSCs (*n* = 3) and UCM-MSCs (*n* = 3). **b** Clustering of the samples based on distance according to RNA-seq analysis. The samples clustered origin-related. **c** The number of differentially expressed genes between AM-MSCs and UCM-MSCs represented by a diagram. A total of 2100 genes is more highly expressed in AM-MSCs and 1857 genes relatively low expressed. **d** GO-term enrichment analysis, based on biological process (BP), of genes, differentially expressed between AM-MSCs and UCM-MSCs. Significantly highly expressed genes in AM-MSCs are highlighted in red while blue represents DEGs in UCM-MSCs. **e** RT-qPCR analysis of pluripotency-related genes, *OCT4, NANOG,* and *SOX2*. **f** RT-qPCR analysis of inflammatory-related genes, *IL-1β, TNF-α, IL-6, TGFβ1,* and *IL-10*. The highly expressed genes *TNF-α,* and *IL-6* are also categorized as stemness-related genes. *GAPDH* was used as an internal control. *P*-values < 0.05 were considered significant. *, *P* < 0.05; **, *P* < 0.01; ***, *P* < 0.001

Next, we compared the global transcriptomes of each 3 AM-MSC and UCM-MSC lines derived from different individuals. A total of 3957 genes were differentially expressed between the two stem cell groups (Fig. 2a). Because AM-MSCs and UCM-MSCs were different origins, they had different gene profiles (Fig. 2b). Among them, 2100 genes were significantly more highly expressed in AM-MSCs and 1857 genes were more highly expressed in UCM-MSCs (Fig. 2c). We also performed GO-term enrichment analysis to classify the genes with similar genetic functions in the biological process. The result showed that the genes differentially expressed between the two types of stem cells fell into several different biological categories, such as biological regulation, metabolic process, response to stimulus, signaling, development process, immune system process, proliferation, reproductive process, and growth (Fig. 2d).

In quantitative PCR for gene expression, it showed that AM-MSCs expressed higher levels of representative stem cell makers, octamer-binding transcription factor 4 (*OCT4*), *NANOG*, and sex-determining region Y-box 2 (*SOX2*) (Fig. 2e, *P < 0.01*). These results suggested that AM-MSCs had more stemness potential and were promising for further research [30]. In terms of inflammation-related genes, expression of *IL-1β* was significantly lower, while that of tumor necrosis factor-alpha (*TNF-α*)*, IL-6,* transforming growth factor-beta (*TGFβ*)*,* and *IL-10* was higher in all three AM-MSC preparations than in the UCM-MSCs (Fig. 2f, *P < 0.01*). IL-1β is a pro-inflammatory cytokine involved in the acute inflammatory response and graft death after transplantation [31]. The lower levels of expression of *IL-1β* and higher levels of *TGF-β* and *IL-10*, which are known as anti-inflammatory cytokines, could reduce inflammatory responses when AM-MSCs are transplanted into patients.

In summary, AM-MSCs and UCM-MSCs are similar in morphology and expression of surface markers but differ in size and gene expression.

### The mass-production potential of AM-MSCs

**Fig. 3:**
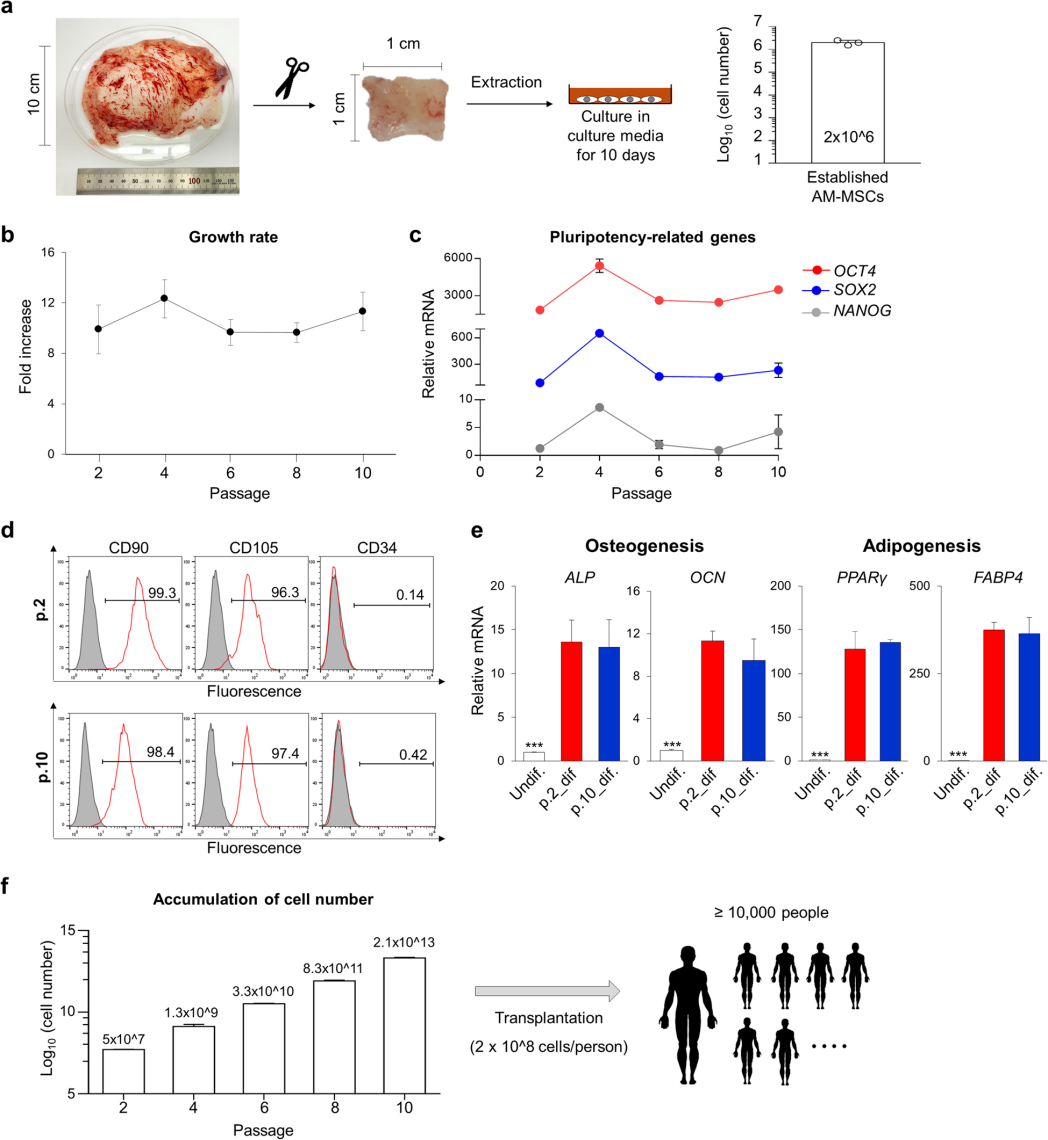
Mass production of AM-MSCs. **a** The isolation procedure, and the number of established AM-MSCs per cm^2^ of the amniotic membrane. Y-axis values represent the logarithm of total cell numbers. **b** Growth rate of AM-MSCs based on passages (Biological replication, *n* = 3). **c** mRNA levels of pluripotency-related genes *OCT4*, *SOX2,* and *NANOG* according to the passage number. *GAPDH* was used as an internal control for RT-qPCR (Technical replication, *n* = 3). **d** Expression percentage of MSC markers (CD90, CD105) and hematopoietic markers (CD34) in early (p.2) and late (p.10) passage of AM-MSCs. **e** mRNA expression analysis of osteogenesis-related (*ALP, OCN*) and adipogenesis-related (*PPARγ, FABP4*) genes on day 14 after the induction of differentiation (Biological replication, *n* = 3). *GAPDH* was used as an internal control. P-values < 0.05 were considered significant. *** *P* < 0.001. undif, undifferentiated cells; dif, differentiated cells. **f** Estimation of total cell numbers during the passage. More than 2 × 10^13^ AM-MSCs could be harvested after ten passages. If maximally, the 2 × 10^8^ cells are used to transplant into a patient, it can be used in more than 10,000 people. Y-axis values represent the logarithm of total cell numbers

For applications as cell-based therapies, large-scale expansion of stem cells is required. To test the mass-production potential of AM-MSCs, we counted the number of stem cells isolated per cm^2^ of the amniotic membrane (Fig. 3a). In three independent experiments, the average number of AM-MSCs has harvested 2 × 10^6^ cells per cm^2^. These stem cells were grown at least 8-fold at every passage up to 10-passage (Fig. 3b).

To confirm that AM-MSCs maintain their characteristics until passage 10, first, we measured the mRNA expression of pluripotent markers [32], *OCT4, NANOG,* and *SOX2,* in every other passage. All three genes were expressed at similar levels among passages (Fig. 3c). Second, we measured the MSC CD marker’s expressions and compared passages 2 and 10 resulting in similar patterns (Fig. 3d). More than 95% of CD90 and CD105 were expressed while CD34 was not expressed in both passages. Finally, we examined the mesenchymal lineage differentiation ability and conducted osteogenic and adipogenic differentiation using passages 2 and 10 of AM-MSCs. Both passages were highly expressed than undifferentiated AM-MSCs in terms of the osteogenic markers; *ALP* and *OCN* and adipogenic markers; *PPARγ* and *FABP4* (*P < 0.001,* Fig. 3e). Between 2 passages, the levels were comparable, suggesting that AM-MSCs maintained their differentiation ability at passage 10.

In conclusion, since the stem cells have shown a growth more than 5 times per passage up to passage 10 while maintaining stemness, more than 10^13^ cells could be obtained from a donor individual (Fig. 3f). Given that a maximum of 2 × 10^8^ cells is used per person in clinical trials of transplantation [33], this yield would be sufficient to treat more than 10,000 individuals.

### Generation of universal cells from AM-MSCs

**Fig. 4:**
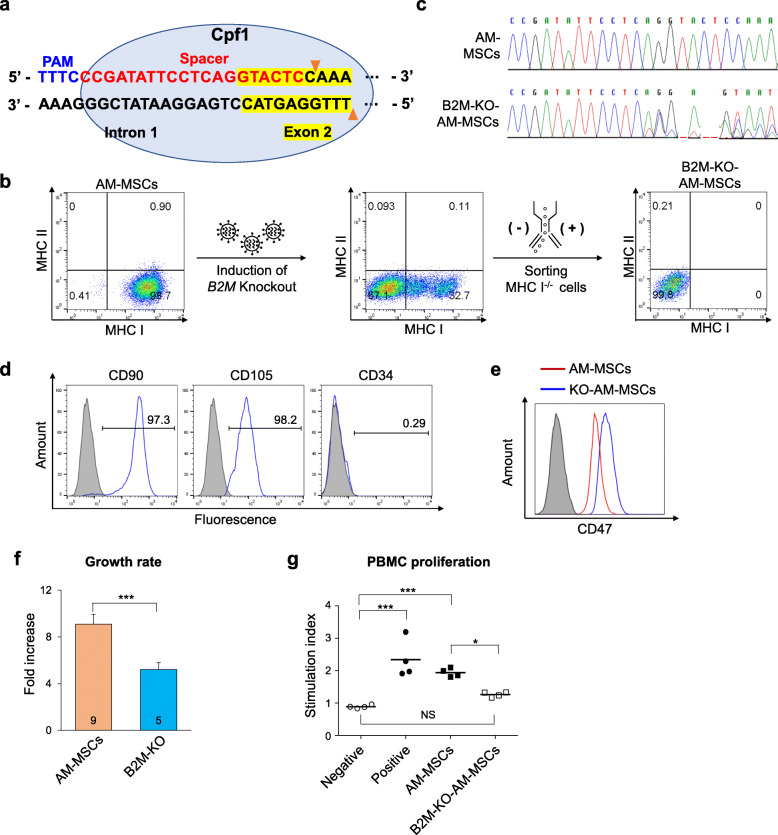
Generation of hypo-immune potential universal cells from AM-MSCs. **a** The target DNA sequence in the human *B2M* locus is shown in red. **b** Schema of the process for generating universal donor AM-MSCs. After inducing B2M knockout, MHC I-negative AM-MSCs are selected by flow cytometry. **c** Sequencing of *B2M* in MHC I-KO and AM-MSCs after B2M knockout in AM-MSCs. **d** Expression of MSC CD markers (CD90, CD105) and hematopoietic markers (CD34) in B2M KO-AM-MSCs. **e** CD47 expression levels in AM-MSCs and B2M-KO AM-MSCs analyzed by flow cytometry. **f** Growth rate of AM-MSCs and B2M-KO-AM-MSCs. B2M-KO: B2M-KO-AM-MSCs. **g** The evaluation of PBMC proliferation assays (4 donors of PBMCs). All control groups were only cultured in a medium without MSC. Negative control: PBMC cultured without PHA, Positive control: PBMC cultured with PHA. NS: no significant, *P*-values < 0.05 were considered significant; *, *P* < 0.05; **, *P* < 0.01; ***, *P* < 0.001

We also induced B2M-knocking out on AM-MSCs to show potential as universal donors. To generate B2M-KO-MSCs, we targeted the *B2M* gene, which up-regulates MHC I, using the CRISPR-Cpf1 system (Fig. 4a) [34]. Stem cells, including AM-MSCs, are MHC I positive, and so can cause immune rejection in transplanted recipients. After inducing B2M-knockout (KO), MHC I-negative cells were sorted by flow cytometry. As we expected, the expression of MHC I was declined, and the B2M gene was successfully knockout (Fig. 4b and c). The KO-AM-MSCs have measured the expression of MSC CD markers. The CD90 and CD105 expressed positively while CD34 was negative (Fig. 4d), but the growth rate was decreased in the B2M-KO (Fig. 4f). However, interestingly, CD47 expression was up-regulated than intact AM-MSCs (Fig. 4e). The CD47 protects transplanted cells from macrophage phagocytosis. This is because when CD47 expression is low, macrophages can recognize the transplanted cells as non-self, known as the *do not eat me signal* [35]. Finally, we evaluated the immune response of B2M-KO-AM-MSCs with human PBMCs using a proliferation assay. The B2M-KO-AM-MSCs were comparable proliferation with negative control while significantly lower than intact AM-MSCs (*P < 0.05*, Fig. 4g) suggesting less immune response.

### In vitro differentiation of AM-MSCs into hepatic progenitors

**Fig. 5:**
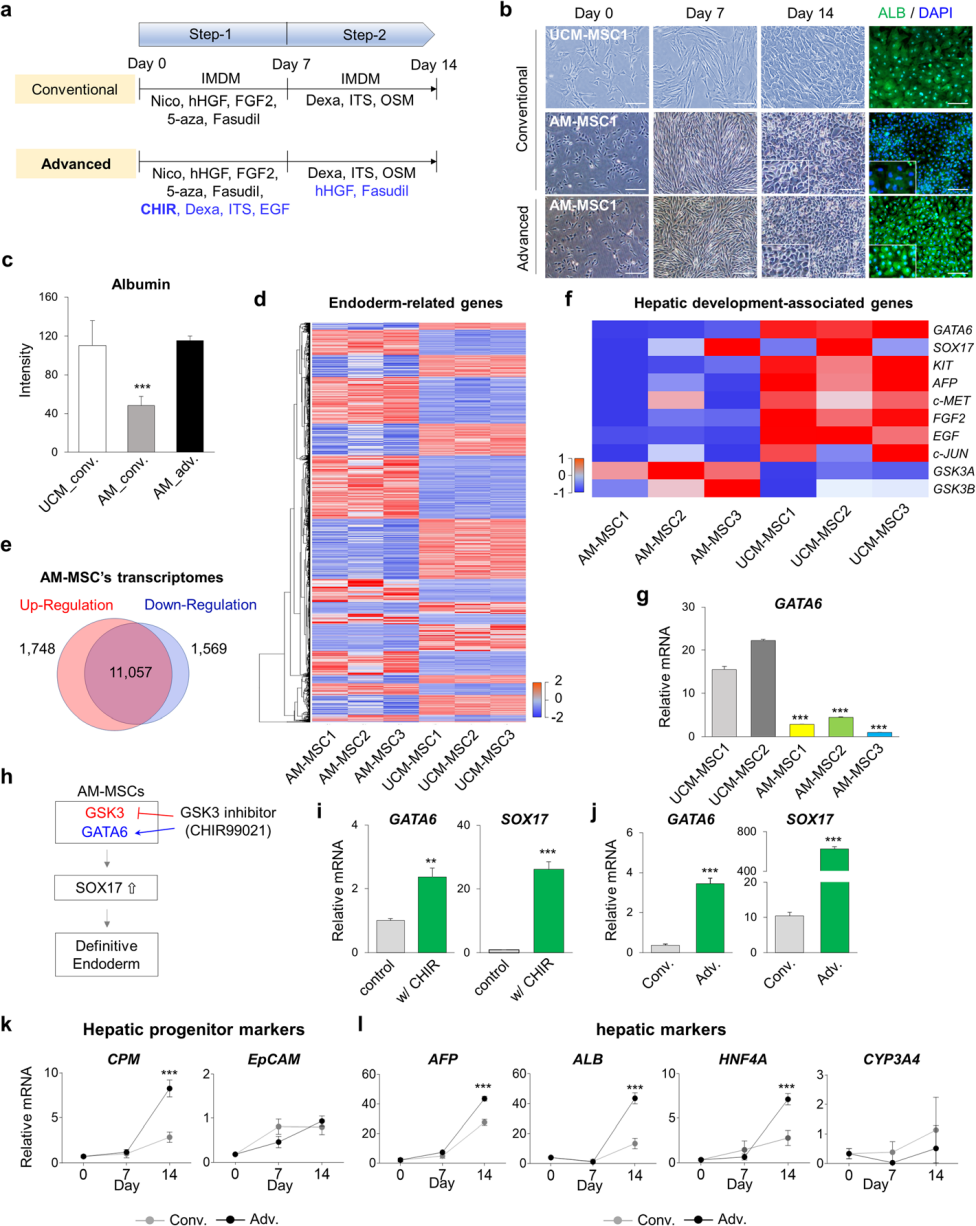
An efficient protocol for enhancing the hepatic differentiation potential of AM-MSCs. **a** The conventional and advanced in vitro hepatic differentiation protocols. The advanced protocol was modified from the conventional protocol by adding several chemicals highlighted in blue. OSM: Oncostatin M. **b** Morphology of cells during hepatic differentiation and ALB expression on day 14. Green, ALB; Blue, DAPI. Scale bar = 200 μm. **c** Fluorescence intensity of detected albumin in (**b**). **d** Hierarchical cluster analysis, and heatmap of endoderm-related transcriptomes in AM-MSCs (*n* = 3) and UCM-MSCs (*n* = 3). **e** Numbers of endoderm-related genes differentially expressed between AM-MSCs and UCM-MSCs. **f** Heat map of hepatic development-associated gene expression in AM-MSCs (*n* = 3) and UCM-MSCs (*n* = 3). **g** RT-qPCR analysis of *GATA6* in AM-MSCs and UCM-MSCs. **h** Scheme of the effect of GSK3 inhibitor (CHIR99021) on AM-MSCs. **i** Regulation of *GATA6,* and *SOX17* in AM-MSCs in the presence of CHIR99021. **j** Effect of the different protocol on the regulation of *GATA6* and *SOX17* in AM-MSCs. **k** RT-qPCR analysis of selected hepatic progenitor markers. **l** Expression of early hepatic (*AFP*) and hepatic maturation (*ALB,* and *HNF4A*) markers. *GAPDH* was used as an internal control for RT-qPCR. Two-way analysis of variance (ANOVA) was performed on the data for differentiation days 0, 7, and 14 points. *P*-values < 0.05 were considered significant. *** *P* < 0.001. Conv. Conventional protocol; Adv. Advanced protocol

In our previous study of hepatic differentiation, MSCs were differentiated into hepatocyte-like cells (HLCs) using specific growth factors or chemicals such as hHGF, FGF2, and 5-azacytidine (5-aza), Fasudil [9]. We attempted to differentiate AM-MSCs into hepatic progenitor cells (HPCs) using the previous conventional protocol (Fig. 5a). The morphological change from an oval or polygonal-like structure was observed (Fig. 5b). However, human albumin (ALB) was significantly lower in AM-MSCs-derived HPCs (AM-HPCs) compared to UCM-MSCs-derived HPCs (Fig. 5c).

To establish an efficient method of hepatic differentiation, we compared gene expression related to the endodermal lineage in AM-MSCs and UCM-MSCs in the previous RNA sequencing data (Fig. 5d). A total of 3317 endoderm-related genes were differentially expressed between the two stem cell types: 1748 were more highly expressed in AM-MSCs, and 1569 in UCM-MSCs (Fig. 5e). We also analyzed the transcription profiles of hepatic development-associated genes (Fig. 5f and Table S2). It showed that *GATA6, KIT, AFP, c-MET, FGF2, EGF, and c-JUN* were down-regulated and *GSK3A* was up-regulated in AM-MSCs (*P < 0.05*). The high levels of GSK3 could repress the induction of definitive endoderm [36]. Transcriptome analysis of AM-MSC and UCM-MSC lines by quantitative PCR confirmed that expression *of GATA6* was much lower in all three AM-MSC lines than in UCM-MSCs (*P < 0.001,* Fig. 5g).

CHIR99021 (CHIR), also known as GSK3 inhibitor, could induce GATA6 expression for hepatic differentiation from human embryonic stem cells [37]. Also, down-regulated *GSK3* could induce upregulation of *GATA6* in cancer cells [38]. Therefore, we hypothesized that inhibition of GSK3 could increase the expression of *GATA6* (Fig. 5h). CHIR was treated for 2 days and measured the expression of *GATA6,* and *SOX17,* which is known as the definitive endodermal marker. As we expected, these two genes showed significantly increased expression in the w/CHIR group than control (*P < 0.01,* Fig. 5i).

Next, we investigated the effect of adding various factors along with CHIR to increase the expression of *GATA6* and *SOX17*. Epidermal growth factor (EGF) and dexamethasone (Dexa) play an important role in hepatic biology and maturation [39]. Insulin-transferrin-selenite (ITS) and fasudil also enhance the stability of stem cell cultures and their differentiation [40]. Based on these factors, we modified the conventional to the advanced protocol by adding CHIR, Dexa, ITS, and EGF. In response, the expression of *GATA6* and *SOX17* was significantly increased on day 2 (*P < 0.001,* Fig. 5j). Therefore, we efficiently and successfully induced definitive endoderm for hepatic differentiation. We also added hHGF and fasudil in step 2 (Fig. 5a). Finally, we observed cell morphology and stained it for ALB expression. The oval or polygonal-like structure of the cells was similar, but ALB was more highly expressed on day 14 (Fig. 5b and c). These findings indicate that the GSK3 inhibitor, CHIR, is essential to induce hepatic differentiation of AM-MSCs through the GATA6 signaling pathway.

We also analyzed the kinetics of gene expressions during HPC differentiation. The expression of *CPM* increased significantly on day 14 in the advanced protocol, while the expression of *EpCAM* was similarly expressed (*P < 0.001*, Fig. 5k). Alpha-fetoprotein (*AFP),* as early hepatic marker, *ALB* and hepatocyte nuclear factor 4 alpha (*HNF4A),* as markers of hepatic maturation, were more highly expressed on day 14 in the advanced protocol (*P < 0.001*, Fig. 5l), while the expression of cytochrome P450 3A4 (*CYP3A4)* was comparable.

### The use of PVA for inducing hepatic differentiation

**Fig. 6:**
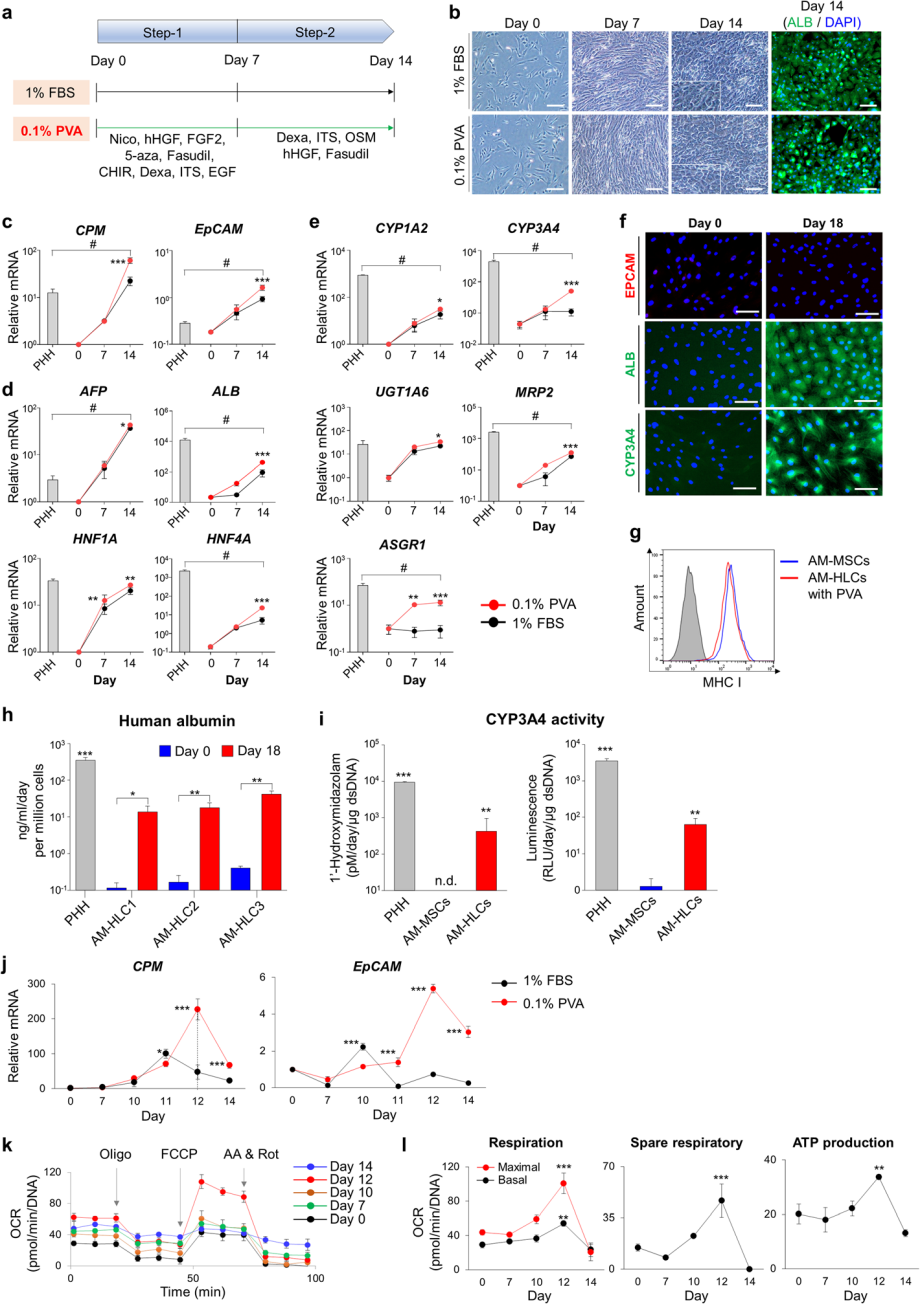
Comparison of PVA and FBS on hepatic differentiation. **a** Scheme for hepatic differentiation using 0.1% PVA or 1% FBS in the advanced protocol. OSM: Oncostatin M. **b** Comparison of morphological change during the differentiation when used 0.1% PVA or 10% FBS. Scale bar = 200 μm. **c-e** The mRNA levels of **c** hepatic progenitor markers (*CPM* and *EpCAM*), **d** liver-specific plasma and nuclear protein (*AFP, ALB, HNF1A,* and *HNF4A*), and **e** liver-specific metabolism (*CYP1A2, CYP3A4, UGT1A6, MRP2,* and *ASGR1*) in primary human hepatocytes (PHH), PVA, and FBS groups. *GAPDH* was used as an internal control. #, *P* < 0.05. **f** The expression of EpCAM, ALB, and CYP3A4 in day 0 (undifferentiated cells) and differentiated cells (hepatocyte-like cells; HLCs) on day 18 after the induction of differentiation. Green, ALB and CYP3A4; Red, EpCAM; Blue, DAPI. Scale bar = 100 μm. **g** Comparison of MHC class I expression between AM-MSCs and AM-HLCs with PVA. **h** Detection of human albumin secretion in PHH and AM-HLCs, which was derived from three independent AM-MSCs. Day 0 were indicated undifferentiated cells, and Day 18 were indicated differentiated HLCs. **i** Measurement of CYP3A4 activity using two experiments: Left, detection of 1-Hydroxymidazolam using LC-ESI/MS/MS systems; Right, detection of luciferin density using luminescence systems (PHH: Technical replicate, *n* = 3; AM-MSCs and AM-HLCs: Biological replicate, *n* = 3). n.d., non-detected **j** Time-dependent comparison of effects on *CPM* and *EpCAM* expression during hepatic differentiation. **k** Oxygen consumption rates (OCR) according to the day of hepatic differentiation. Olig: oligomycin, FCCP: carbonyl cyanide 4-(trifluoromethoxy) phenylhydrazone, AA: antimycin A, Rot: rotenone. **l** Changes of OCR values of basal and maximal respiration, spare respiratory capacity, and ATP production. OCR values were normalized by the DNA concentration. *P*-values < 0.05 were considered significant. *, *P* < 0.05; **, *P* < 0.01; ***, *P* < 0.001

FBS is still widely used for inducing hepatic differentiation [41]. However, it causes an acute immune response after transplantation because of xeno-contamination. Therefore, we substituted 0.1% PVA for 1% FBS in the advanced protocol (Fig. 6a). The phenotype of differentiated cells was similar when both PVA and FBS were used (Fig. 6b). First, we analyzed the dynamics of gene expressions associated with hepatocyte-related genes on days 7 and 14 using previous reports [42, 43], and compared them with primary human hepatocytes (PHH) control. *CPM* and *EpCAM*, which are related to hepatic progenitor markers, were expressed more highly in the PVA group on day 14 (*P < 0.01*, Fig. 6c). We also analyzed the genes, which are associated with liver-specific plasma and nuclear protein such as *AFP, ALB, HNF1A,* and *HNF4A*. The levels of expression of *AFP, ALB, HNF1A*, and *HNF4A* also were higher in the PVA group on day 14 (*P < 0.01*, Fig. 6d). Moreover, *CYP1A2, CYP3A4, UGT1A6, MRP2,* and *ASGR1*, which are related to liver-specific metabolism, were expressed higher in the PVA group on day 14 (*P < 0.05*, Fig. 6e). Compared with primary hepatocytes, PHH was a significantly lower expression of *CPM, EpCAM*, and *AFP* (*P < 0.05*), while *HNF1A* and *UGT1A6* were comparable. The other genes were higher than on day 14 of the PVA group (*P < 0.05*).

Moreover, we applied the PVA-advanced protocol to UCM-MSCs. The efficiency of hepatic differentiation was significantly increased than the conventional one (Fig. S1a). We also reanalyzed and compared the previously published data [9], the AM-HPCs were more similar to PHH than those derived from either UCM-MSCs or LD-SCs (Fig. S1b).

Next, we examined the protein expression of ALB, EpCAM, and CYP3A4 to evaluate differentiation into HLC in PVA-used protocol. At 18 days after differentiation, the mature markers, ALB, and CYP3A4 were successfully expressed, while the hepatic progenitor marker, EpCAM, was declined (Fig. 6f). These differentiated HLCs showed MHC I expression similar to intact AM-MSCs (Fig. 6g).

Finally, because the main functions of hepatocytes are protein synthesis and detoxification, we performed to measure albumin secretion and CYP3A4 activity in media cultured AM-HLCs. We confirmed that the secretion of human albumin was significantly increased on day 18 of AM-HLCs than day 0 of undifferentiated cells, but less secreted than PHH control (*P < 0.05,* Fig. 6h). The CYP3A4 activity was measured by two independent methods. First, we treated the substrate of CYP3A4, midazolam, to PHH, AM-MSCs, and AM-HLCs for 24 h and measured the metabolite of midazolam, 1-hydroxymidazolam, using LC-ESI/MS/MS system [26]. The metabolite was detected in PHH and AM-HLCs groups (Fig. 6i and Fig. S2). Next, we measured the enzyme activity of AM-HLCs using the substrate-luminescence systems. The luminescence was also increased in the PHH and AM-HLCs groups compared with undifferentiated cells (*P < 0.01,* Fig. 6i). But, the activity of the enzyme in AM-HLCs was shown to a lower than PHHs (*P < 0.001*). These results suggest that AM-HPCs with PVA are useful for transplantation into diseased livers because the cells produced albumin and CYP3A4 enzyme, which could catalyze toxic drugs in the liver [44].

Bipotent hepatic progenitor cells provide more efficient rehabilitation than mature hepatocytes after transplantation [45]. We have reported that day 14 after hepatic differentiation is the best time for transplanting UCM or LD hepatic progenitors [9]. To decide a suitable transplantation time for the AM-HPCs in vivo, we conducted two experiments to examine the functional properties of the HPCs. First, we investigated the highest expression date of *CPM* and *EpCAM*, which are critical markers for bi-potential hepatic progenitor cells that can be differentiated into hepatocytes or cholangiocytes [43]. The *CPM* was most highly expressed in the FBS group on day 11, while in the PVA group it was highest on day 12 (*P < 0.01,* Fig. 6j). The *EpCAM* was most highly expressed on days 10 and 12 in the FBS and PVA groups, respectively (*P < 0.001,* Fig. 6j). Second, we observed mitochondrial oxygen consumption rates (OCR) on days 7, 10, 12, and 14 following differentiation using PVA. Overall OCR levels were highest on day 12 (Fig. 6k); more specifically, basal and maximal respiration, spare respiratory capacity, and ATP production were significantly elevated on day 12 (*P < 0.01,* Fig. 6l). Spare respiratory capacity is the extra-mitochondrial capacity, which is available under conditions of increased work or stress. Aso, it is considered important for long-term cell survival and function [46]. In conclusion, when PVA is used in hepatic differentiation for xeno-free conditions, it helped increase AM-MSCs maturation into hepatocytes*,* and mitochondrial functions to support their transplantation efficiency.

### In vivo regeneration potential of AM-hepatic progenitor cells

The AM-HPCs were transplanted into the mouse liver failure model to see whether the cells could engraft into functional hepatocytes in vivo. To start with, the protocol to induce liver damage in mice was modified with the inclusion of cyclophosphamide monohydrate (CTX) along with thioacetamide (TAA). TAA is widely used in mice to induce liver failure. However, it requires close monitoring and supportive care of the mice due to lethality before research start [47]. In our previous work, half the mice that received the highest doses (0.2–0.4 mg/g) of TAA died before the start of the study, and the mice were not well-induced at lower doses. Therefore, we needed a more stable mouse model for transplantation studies.

**Fig. 7:**
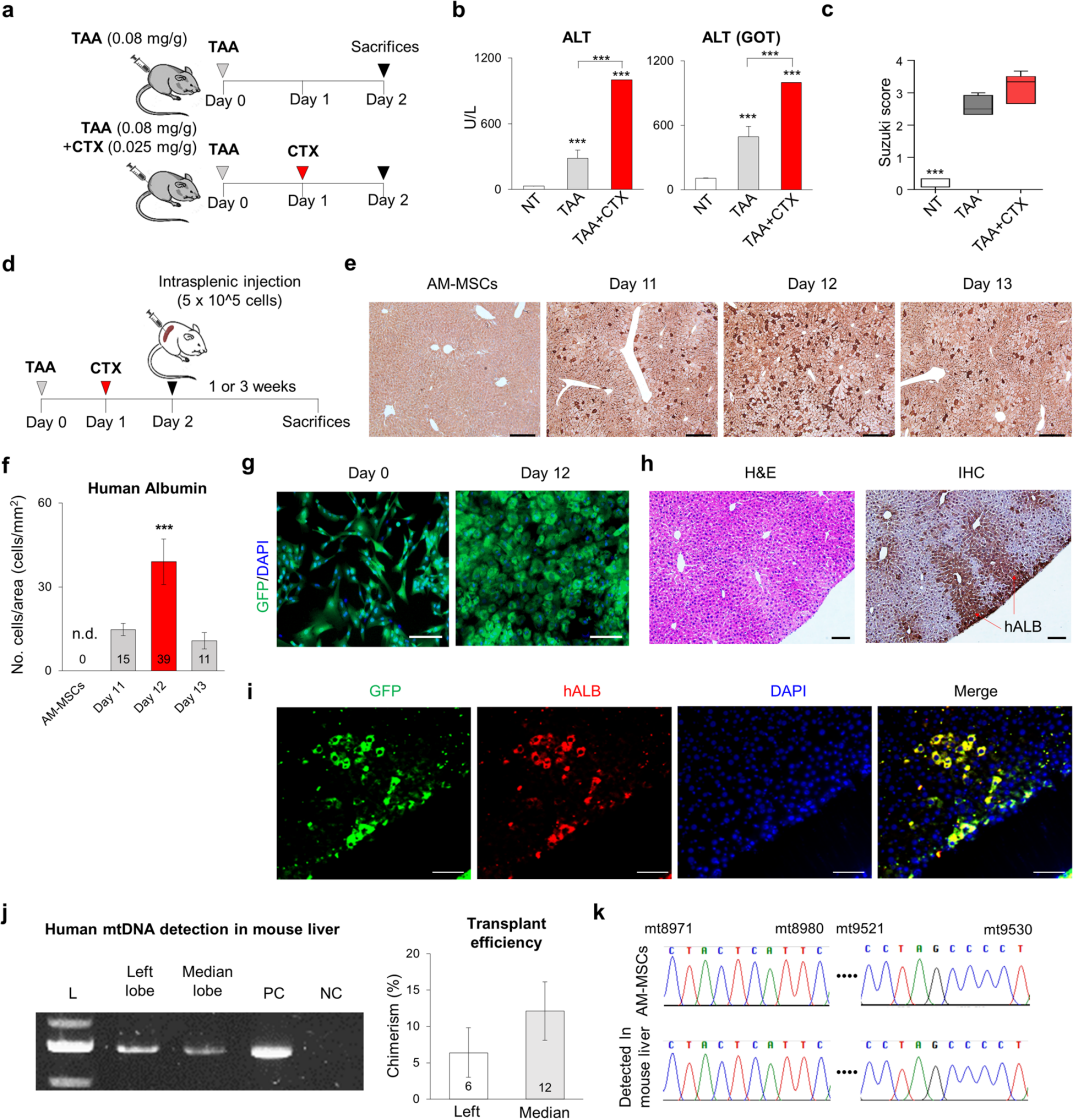
Liver regeneration with AM-MSC-derived HPCs in vivo*.* **a** Schematic summary of the acute liver failure mice model. CTX was injected intraperitoneally 24 h after TAA injection, and blood was collected 24 h later. **b** Levels of ALT and AST-GOT in NT, TAA, and TAA + CTX treated group’s serum. *n* = 4 for NT; *n* = 4 for the TAA group; *n* = 5 for the TAA + CTX group. NT: non-treated groups **c** Suzuki scores of hematoxylin and eosin (HE) staining in NT, TAA, and TAA + CTX treated groups. **d** Schematic summary of transplantation into acute liver failure-inducted NOD-SCID IL2Rgammanull (NSG) mice. **e** Representative immunohistochemistry (IHC) images of human albumin in the mouse liver. The transplanted cells were AM-MSCs and AM-HPCs with differentiation days 11, 12, and 13. Scale bar = 50 μm. **f** Number of human albumin-positive cells per area in Fig. 6e. n.d.: non-detected. (*n* = 4). **g** GFP-positive transplanted cells. GFP was continuously expressed in AM-HPCs. Scale bar = 200 μm. **h** H&E staining, and human albumin level by immunohistochemistry in mouse liver 3 weeks after transplantation. Scale bar = 50 μm. **i** Confocal images of immunofluorescence staining for human albumin (red) with GFP (green). Nuclei were counterstained with DAPI (blue). Scale bar = 200 μm **j** Detection of human mitochondrial DNA in the mouse liver. Left, DNA gel image. Right, Measurement of transplant efficiency using quantitative PCR with mouse and human mtDNA calibration curve. *GAPDH* was used as an internal control. PC, human positive control; NC, negative control. **k** Human mtDNA Sanger sequencing in AM-HPCs and mouse liver. *P*-values < 0.05 were considered significant. *, *P* < 0.05; **, *P* < 0.01; ***, *P* < 0.001

CTX is also toxic to the bone marrow and liver tissue [48]. Based on our previous work, we devised an improved acute liver failure model using 0.025 mg/g CTX and 0.08 mg/g TAA (Fig. 7a). First, we measured the survival rate of the mouse model. A total of 11 mice were inducted into the liver failure model. Two died on day 1, four died on day 2, and five died on day 3 after induction (Fig. S3b). On day 1 after induction, whole blood was collected from the mouse model and analyzed complete blood counts (CBC) and blood enzyme analyses. The markers for liver damage, alanine aminotransferase (ALT), and aspartate aminotransferase (AST-GOT), and the percentages of granulocytes and lymphocytes were significantly increased in CTX + TAA treated groups compared with the untreated control (*P < 0.001*, Fig. 7b; and *P < 0.05,* Table S3). Furthermore, both ALT and AST were significantly more highly expressed in the CTX + TAA group compared with the TAA-only model, while the percentages of granulocytes and lymphocytes were similar liver damage occurred around the hepatic vein during the initial exposure of the liver to the drugs (Fig. S3a). The modified model had a similar effect on acute liver failure as the TAA model, but the Suzuki score tended to be higher (Fig. 7c), and the latter appears to be the historical criterion for assessing liver injury [27]. In conclusion, we decided that cells could transplant 1 day after the induction of the liver failure model, the same as 2 days from the first induction (Fig. 7d).

Finally, to confirm the therapeutic capacity and engraftment of AM-HPCs, we transplanted half a million of AM-MSCs and AM-HPCs on different days 11, 12, and 13 into the spleens of the NSG mouse model. One or 3 weeks later, the mice were examined (Fig. 7d). Most of the induced model mice were survival before sacrificing and the histological images of the liver were displayed normal morphology, suggesting that both AM-MSCs and AM-HPCs had the therapeutic effect (Fig. S3c). We could not evaluate the comparison of therapeutic effects among groups in this study. However, in human ALB expression, on the day 12 AM-HPC group was detected a higher number of albumin positive cells than other differentiated groups, while the intact AM-MSC group was not detected any positive cells (*P < 0.01*, Fig. 7e and f). Thus, all groups included AM-MSCs showed a therapeutic effect including a paracrine effect [49, 50], while as we expected, on day 12 AM-HPCs were a higher engraftment rate with hepatic differentiation in the mouse liver.

To track the transplanted cells, we transfected a green fluorescent protein (GFP) reporter gene to the AM-MSCs and confirmed that the GFP-expressions in AM-MSCs and AM-HPCs (Fig. 6g). Three weeks later, human albumin was detected around the hepatic vein of the mouse liver with histological recovery (Fig. 7h). The ALB-positive cells were co-localized with GFP-expression (Fig. 7i). Additionally, we examined human mtDNA detection in liver tissue. PCR band was shown that there was approximately 6%–12% human mtDNA present in the mouse liver (Fig. 7j). The PCR products were confirmed by Sanger sequencing result in human mtDNA sequence (Fig. 7k and Fig. S3d).

These results suggest that AM-HPCs can successfully engraft in damaged livers and supported regeneration.

## Discussion

**Fig. 8:**
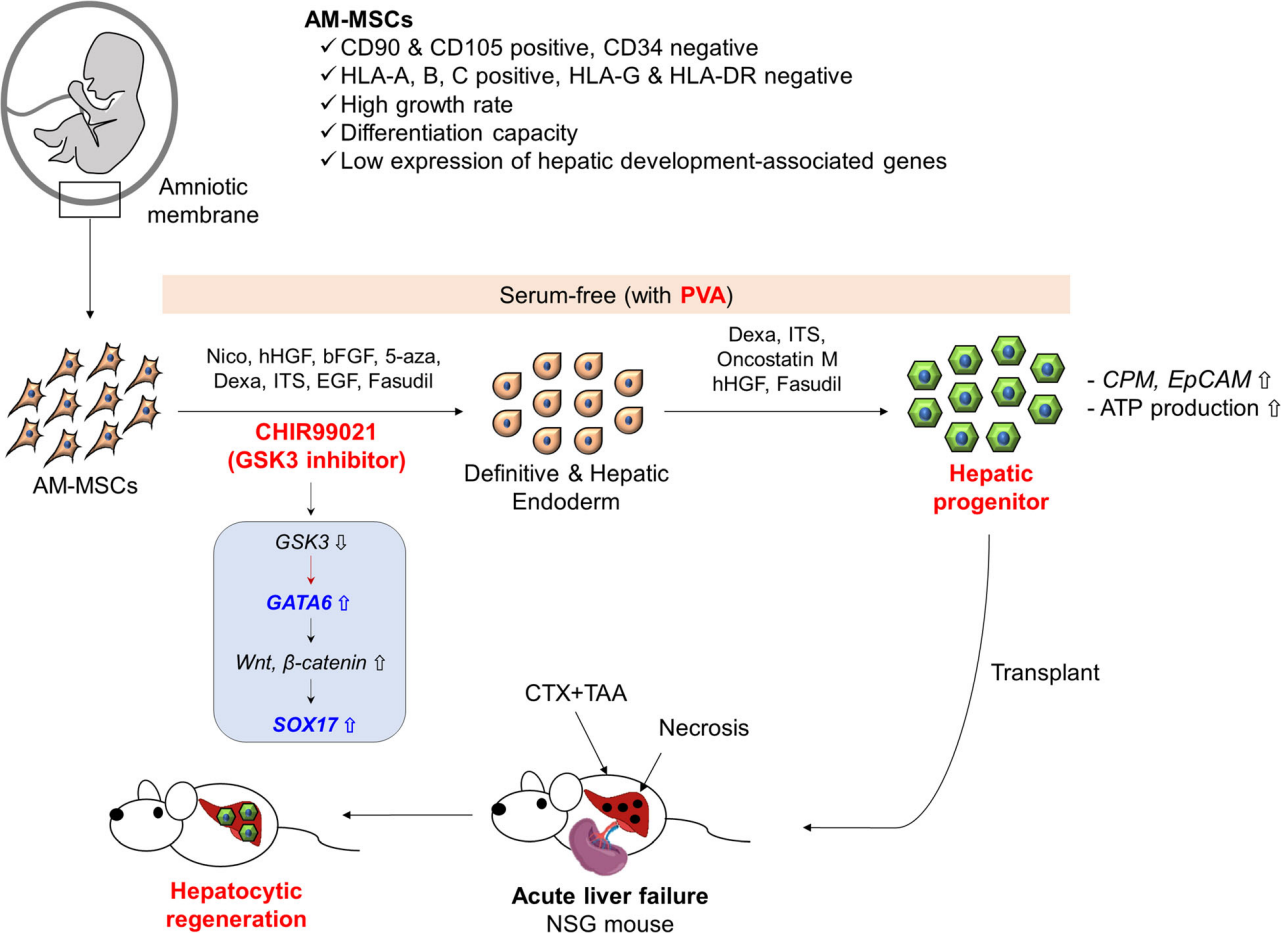
Schematic illustration of the overall flow of this study. AM-MSCs were isolated and characterized. However, they had low levels of *GATA6* and high levels of *GSK3,* which are reversed conditions for inducing definitive endoderm (DE). Accordingly, the efficiency of hepatic differentiation was low when the conventional protocol was used. To increase efficiency, CHIR99021 and several other factors were added to the definitive and hepatic endoderm induction medium. Also, FBS was substituted by PVA to provide xeno-free conditions for transplantation and enhance the efficiency of differentiation. After that, the maturation of hepatocyte-like cells (HLCs) from AM-MSCs (on differentiation day 12; HPCs) was investigated in vivo. For this, we established a two-chemical (CTX + TAA) model of acute liver failure in mice with improved stability of induction compared with the TAA-induced model. The AM-HPCs were successfully transplanted into the liver and spontaneously repopulate in the acute failure liver

We have successfully regenerated hepatocytes in a mouse model using a newly developed method for HPC differentiated from human AM-MSCs (Fig. 8). The advantage of stem cells for cell therapy would be safety, mass-producibility, and the ability to select the most effective cells. The ideal stem cells of the future are iPSCs, but there remain issues of safety and insufficient differentiation still exist [51]. The various stem cells derived from neonatal-related tissues differ in yields and differentiation abilities depending on the origin of the tissue and desired differentiated cell type. AM-MSCs are suitable for mass production because of the large amount of original tissue material. Based on our data, if passage 10 is used for therapy, the AM-MSCs established from a single individual could be transplanted into more than 10,000 patients without change of characteristics.

We isolated the AM-MSCs from the amniotic membrane, which generally was isolated epithelial cells [52]. For obtaining amniotic epithelial cells, a collagen-coating plate or EGF supplement was needed. We only used 10% FBS supplemented with bFGF, which could support MSCs characteristics. They were expressed almost 95% CD105 positive and negative HLA-G (MHC class 1b) [53]. MHC class 1b is known as an expression in amnion derived-epithelial cells [29]. Also, EpCAM, which is a positive expression in the epithelium derived from the anionic membrane and hepatic progenitor cells [53, 54], was rarely expressed in AM-MSCs.

AM-MSCs can be isolated non-invasively like neonatal-related tissues, and easily banked depending on their characteristics. AM-MSCs did not express MHC class II and HLA-G but did express HLA-A, B, and C. MHC class I and class II antigens, corresponding to HLA-A, B, C, G, and HLA-DP, DM, DO, DQ, and DR, respectively, have major impacts on graft failure [55]. Matching HLA type or inhibiting MHC expression can reduce or eliminate the use of immunosuppressive drugs, which are unpleasant and can cause side effects [56]. However, additional molecules, such as PD-L1, HLA-E, and CD47, have also been recently identified supporting and describing the lack of immunorecognition upon transplantation in allogenic and xenogenic settings [57]. Therefore, AM-MSCs can be candidate cells for universal donors that have applied gene-editing technology.

The next major hurdle was that somatic stem cells did not have sufficient ability to differentiate into HPCs or HLCs. AM-MSCs had similar characteristics to UCM-MSCs in terms of phenotype but differed in gene expression. Thus, we realized that different differentiation protocols might be necessary depending on the origin of the cells. UCM-MSCs was previously reported that the cells expressed hepatocyte-related genes and could easily differentiate into HLCs [58]. Moreover, because of their similar characteristics, UCM-MSCs can be the comparative control of AM-MSC’s transcriptome analysis. As a result, the AM-MSCs have lower expression levels of endoderm transcription factors, such as *GATA6, KIT, AFP, c-MET, FGF2, EGF, and c-JUN,* and higher expression levels of *GSK3*.

Because CHIR has been previously used for hepatic differentiation from PSCs [59], we hypothesized that the GSK-3 inhibitor, CHIR, might promote differentiation to HPCs from AM-MSCs. This study showed that CHIR induced the expression of GATA6 and SOX17, which were necessary genes for stem cells to differentiate into hepatocytes. Probably, GSK3 inhibited by CHIR activated GATA6 signaling, which upregulated the Wnt/β-catenin pathway, leading to the enhancement of the *SOX17* gene [38, 60, 61] (Fig. 8). Therefore, CHIR99021 with the addition of supplements, the advanced protocol conferred robust differentiation ability on the AM-MSCs.

Besides, the ROCK inhibitor, Y-27632, has been used for differentiating hiPSCs to HPCs and reprogramming mature hepatocytes to the HPCs [62, 63]. Fasudil is a known ROCK inhibitor and only approval for clinical and it is similar to Y-27632 for using cell culture and differentiation [40]. Thus, we applied fasudil for differentiating AM-MSCs. The other growth factors and chemicals such as EGF, FGF2, HGF, Dexa, ITS, Nico, 5-aza, and OSM might promote the differentiation of definitive endoderm into hepatic endoderm or hepatic progenitor cells based on previous reports (Table S4).

FBS can contaminate cells during cell culture and differentiation, resulting in the presence of xeno-antigens and infectious agents that can provoke graft-versus-host disease [64]. It can also lead to differences between batches [65]. Hence, FBS must be replaced with an alternative supplement to achieve therapeutic outcomes in patients, but its use is still popular for hepatic differentiation despite the risks associated with transplantation [41]. Polyvinyl alcohol (PVA) is a water-soluble synthetic polymer and has been used as a substitute for bovine serum albumin (BSA) in culture media for mouse preimplantation embryos [66]. It is also used in various human cell expansion and differentiation procedures for therapeutic applications, including hepatic differentiation from PSCs [67–70]. Interestingly, PVA could not only replace FBS but also significantly improved the differentiation to HPCs of AM-MSCs.

As in vivo study models, we induced two chemicals (TAA and CTX) to cause liver failure in mice. In previous studies, TAA is widely used in hepatic disease studies in models. However, TAA-induced models have difficulties controlling because of side effects [47]. For this reason, mouse models were easily dead when TAA was used. In addition, the model was not well induced in a low dose of TAA. Therefore, we used CTX, which is also known as toxic to the liver, and expected that CTX can be affected by the stability and liver damage of the mouse model. Our studies showed that TAA with CTX-induced mice has significantly increased expression of the liver damage markers (ALT, AST-GOT), and successfully induced necrosis on mouse liver.

To identify optimal transplanting cells, we transplanted AM-MSCs, day 11, day 12, and day 13-differentiated cells into the mouse model. Unlike previous reports with BM-MSCs or UCM-MSCs that can differentiate into hepatocyte-like cells in vivo [71, 72], AM-MSCs could not be differentiated in vivo. Probably, since AM-MSCs require additional small molecules such as CHIR99021 for hepatic differentiation in vitro, the cells mainly contributed to supporting the regeneration of damaged liver with mouse’s cells instead of differentiation. Even though GSK3 inhibitors were used in a pre-clinical and clinical study for treating cancer [73], it may not be an efficient method to use AM-MSCs along with GSK3 inhibitors *for* in vivo differentiation due to the side effect or dosage of chemicals [74, 75]. On day 12, differentiated cells were the best conditions in terms of the number of human albumin-positive cells in the mouse liver. As we expected this time was matched with higher expression of *CPM* and *EPCAM* and OCR levels of mitochondria suggesting useful as biomarkers for transplanting of HPCs.

## Conclusions

This study established a more effective hepatic differentiation protocol for AM-MSCs by analyzing transcriptome profiles. Moreover, our findings suggested that PVA can be a suitable alternative to FBS in the differentiation for clinical trials. Finally, the results of in vivo experiment-supported AM-HPCs can be repopulated in the acutely injured livers and were spontaneously rescued by liver failure. To sum up, this study highlights the fact that AM-MSC-derived HPCs are a promising resource for treating acute liver failure.

## Supplementary Information

**Additional file 1:** **Table S1.** Primers used in this work. **Table S2.** Information on genes associated with hepatic development. **Table S3.** Complete blood counts and blood enzyme analyses in controls and treated mice. **Table S4.** Sources of somatic stem cells and their outcomes in terms of hepatic differentiation. **Fig. S1** The effect of advanced protocol on UCM-MSCs. **a** Hepatic gene expression was analyzed 14 days after induction of differentiation. **b** Gene expression in day 14 hepatic cells differentiated from different types of adult stem cells; RT-qPCR analysis of selected hepatic differentiation genes (*ALB*, *HMF4A,* and *CPM*) in day 14 cells differentiated from PHH, AM-MSCs, UCM-MSCs, LD-SC1, and LD-SC2. *GAPDH* was used as an internal control for RT-qPCR. *P*-values < 0.05 were considered significant; ns: not significant. *, *P* < 0.05; **, *P* < 0.01; ***, *P* < 0.001. **Fig. S2** Chromatogram of LS-ESI/MS/MS profiles in the culture medium of each group after treating midazolam. Red box: the peak of 1-hydroxymidazolam. **Fig. S3 a** HE-stained tissue from NT, TAA, and TAA + CTX-treated mice. Scale bar = 50 μm. NT: non-treated. **b** Survival rate of CTX + TAA and cell-transplanted groups for 7 days. Number, the number of dead mice. **c** H&E staining images after transplanting AM-MSCs and AM-HPCs differentiated days 11, 12, and 13 in the mouse model. Scale bar = 50 μm. **d** Sanger sequencing of mtDNA from AM-HPCs and liver tissue from the transplanted mouse. Human AM-HPCs were injected into mouse livers and their partial mitochondrial genomes were analyzed by the Sanger sequencing method. The cells had identical mitochondrial DNA sequences. **e** Standard curve of human and mouse mtDNA for calculating transfer efficiency.

## Data Availability

The datasets used and analyzed during the current study are available from the corresponding author on reasonable request.

## Abbreviations

AM-MSCs: Amnion-derived mesenchymal stem cells
UCM-MSCs: Umbilical cord matrix-derived mesenchymal stem cells
HPCs: Hepatic progenitor cells
HLCs: Hepaotycte-like cells
FBS: Fetal bovine serum
PVA: Polyvinyl alcohol
MHC: Major histocompatibility complex
TAA: Thioacetamide
CTX: Cyclophosphamide monohydrate
MSCs: Mesenchymal stem cells
hLD-SCs: Human adult liver-derived stem cells
CPM: Carboxypeptidase M
EpCAM: Epithelial cell adhesion molecule
OCT4: Octamer-binding transcription factor 4
SOX2: Sex-determining region Y-box 2
TNF-α: Tumor necrosis factor-alpha
TGFβ: Transforming growth factor-beta
KO-AM-MSCs: B2M-KO-AM-MSCs
AM-HPCs: AM-MSCs-derived HPCs
UCM-HPCs: UCM-MSCs-derived HPCs
AM-HLCs: AM-MSCs-derived hepatocyte-like cells
CHIR: CHIR99021
ALB: Albumin
AFP: Alpha-fetoprotein
HNF4A: Hepatocyte nuclear factor 4 alpha
HNF1A: Hepatocyte nuclear factor 1 alpha
CYP3A4: Cytochrome P450 3A4
CYP1A2: Cytochrome P450 1A2
UGT1A6: UDP Glucuronosyltransferase Family 1 Member A6
MRP2: Multiple drug resistance-associated protein 2
ASGR1: Asialoglycoprotein receptor 1
OCR: Oxygen consumption rates
ALT: Alanine aminotransferase
AST-GOT: Aspartate aminotransferase
GFP: Green fluorescent protein
