# Supplementary material for: Efficient hepatic differentiation and regeneration potential under xeno-free conditions using mass-producible amnion-derived mesenchymal stem cells
Source: Stem Cell Res Ther. 2021 Nov 12;12:569. doi: 10.1186/s13287-021-02470-y (PMC8588618; doi:10.1186/s13287-021-02470-y)
Supplement: Supplementary file 1 — Additional file 1: Table S1. Primers used in this work. Table S2. Information on genes associated with hepatic development. Table S3. Complete blood counts and blood enzyme analyses in controls and treated mice. Table S4. Sources of somatic stem cells and their outcomes in terms of hepatic differentiation. Fig. S1 The effect of advanced protocol on UCM-MSCs. a Hepatic gene expression was analyzed 14 days after induction of differentiation. b Gene expression in day 14 hepatic cells differentiated from different types of adult stem cells; RT-qPCR analysis of selected hepatic differentiation genes (ALB, HMF4A, and CPM) in day 14 cells differentiated from PHH, AM-MSCs, UCM-MSCs, LD-SC1, and LD-SC2. GAPDH was used as an internal control for RT-qPCR. P-values < 0.05 were considered significant; ns: not significant. *, P < 0.05; **, P < 0.01; ***, P < 0.001. Fig. S2 Chromatogram of LS-ESI/MS/MS profiles in the culture medium of each group after treating midazolam. Red box: the peak of 1-hydroxymidazolam. Fig. S3 a HE-stained tissue from NT, TAA, and TAA + CTX-treated mice. Scale bar = 50 μm. NT: non-treated. b Survival rate of CTX + TAA and cell-transplanted groups for 7 days. Number, the number of dead mice. c H&E staining images after transplanting AM-MSCs and AM-HPCs differentiated days 11, 12, and 13 in the mouse model. Scale bar = 50 μm. d Sanger sequencing of mtDNA from AM-HPCs and liver tissue from the transplanted mouse. Human AM-HPCs were injected into mouse livers and their partial mitochondrial genomes were analyzed by the Sanger sequencing method. The cells had identical mitochondrial DNA sequences. e Standard curve of human and mouse mtDNA for calculating transfer efficiency. [file 13287_2021_2470_MOESM1_ESM.pdf]

Table S1. Primers used in this work

Gene name	Primer sequence (5' to 3')	*F, Forward; R, Reverse
IL-1β	F: AATCTGTACCTGTCCTGCGTGTT<br>R: TGGGTAATTTTTGGGATCTAACTCT	
TNFα	F: TCAGATCATCTTCTCGAACCCC<br>R: ATCTCTCAGCTCCACGCCAT	
IL-6	F: GGAGACTTGCCTGGTGAAAA<br>R: GTCAGGGGTGGTTATTGCAT	
TGFβ1	F: GGCCAGATCCTGTCCAAGC<br>R: GTGGGTTTCCACCATTAGAC	
IL-10	F: GGTTGCCAAGCCTTGTCTGA<br>R: AGGGAGTTCACATGCGCCT	
OCT4	F: GAAGGATGTGGTCCGAGTGT<br>R: GTGAAGTGAGGGCTCCCATA	
NANOG	F: CAAAGGCAAACAACCCACTT<br>R: TCTGCTGGAGGCTGAGGTAT	
SOX2	F: AACCCCAAGATGCACAACTC<br>R: CGGGGCCGGTATTTATAATC	
CPM	F: GGATGGAAGCGTTTTTGAAG<br>R: CCACAACAAGAACCCACAGG	
HNF4A	F: CAGGCTCAAGAAATGCTTCC<br>R: GGCTGCTGTCCTCATAGCTT	
AFP	F: AGACTGCTGCAGCCAAAGTGA<br>R: GTGGGATCGATGCTGGAGTG	
ALB	F: TGCTGATGAGTCAGCTGAAAA<br>R: TCAGCCATTTCACCATAGGTT	
CYP3A4	F: TTTTGTCCTACCATAAGGGCTTT<br>R: CACAGGCTGTTGACCATCAT	
EPCAM	F: GCCAGTGTACTTCAGTTGGTGC<br>R: CCCTTCAGGTTTTGCTCTTCTCC	
UGT1A6	F: GCCCTGTGATTTGGAGAGTGA<br>R: AGGCTTCAAATTCCTGAGACAAGT	
CYP1A2	F: CGGACAGCACTTCCCTGAGA<br>R: AGGCAGGTAGCGAAGGATGG	
MRP2	F: AGCGTCCTCTGACACTCG<br>R: GGCATCTTGGCTTTGACT	
ASGR1	F: CAGCAACTTCACAGCCAGCA<br>R: AGCTGGGACTCTAGCGACTT	
HNF1A	F: TGGGTCCTACGTTCACCAAC<br>R: TCTGCACAGGTGGCATGAGC	
GATA6	F: GAGGCTTGCTGAAAGAGTGAGAGAAGA<br>R: TCCTAGTCCTGGCTTCTGGAAGTG	
SOX17	F: CAAGGGCGAGTCCCGTAT<br>R: CGACTTGCCCAGCATCTT	
GAPDH	F: GCCTCAAGATCATCAGCAATGC<br>R: TGGTCATGAGTCCTTCCACGAT	
B2M (Indel sequencing primer)	F: GCTATGAGTGCTGAGAGGGC<br>R: CACGGCAGGCATACTCATCT	
Human mitochondrial DNA	F: CAACACTAAAGGACGAACCTGA<br>R: TCGTAAGGGGTGGATTTTTC	
ALP	F: ACCATTCCCACGTCTTCACATTT<br>R: AGACATTCTCTCGTTCACCGCC	
OCN	F: CAAAGGTGCAGCCTTTGTGTC<br>R: TCACAGTCCGGATTGAGCTCA	
FABP4	F: GCTTTGCCACCAGGAAAGTG<br>R: ATGGACGCATTCCACCACCA	
PPARγ	F: GATACACTGTCTGCAAACATATCACAA<br>R: CCACGGAGCTGATCCCAA	

Table S2. Information on genes associated with hepatic development

No.	Gene ID	Gene Name	Function	Regulation	Relevance score (from genecards)
1	GATA6	GATA Binding Protein 6	Regulates hepatic fate by acting endoderm-related and hepatic development genes	Enhanced	16.18
2	SOX17	SRY-Box Transcription Factor 17	Necessary for endoderm formation, hepatic development, and hepatocyte differentiation	Enhanced	7.42
3	KIT	KIT Proto-Oncogene, Receptor Tyrosine Kinase	Express in hepatic progenitors. Regulates cell differentiation and proliferation	Enhanced	34.47
4	AFP	Alpha Fetoprotein	Marker of hepatic progenitors and hepatocytes	Enhanced	59.09
5	c-MET	MET Proto-Oncogene, Receptor Tyrosine Kinase	Hepatocyte growth factor (HGF) receptor. It required for hepatocyte survival including liver development	Enhanced	26.27
6	FGF2	Fibroblast Growth Factor 2	Require to specify hepatic fate within the definitive endoderm through activation of the FGF receptors	Enhanced	24.77
7	EGF	Epidermal Growth Factor	It is a necessary ligand for activation of c-JUN	Enhanced	43.52
8	c-JUN	Jun Proto-Oncogene, AP-1 Transcription Factor Subunit	Necessary for hepatic development associated with hepatoblasts	Enhanced	26.27
9	GSK3A	Glycogen Synthase Kinase 3 Alpha	Inhibits the induction of definitive endoderm by repressing c-Myc and Beta-catenin.	Repressed	4.52
10	GSK3B	Glycogen Synthase Kinase 3 Beta		Repressed	20.53

Table S3. Complete blood counts and blood enzyme analyses in controls and treated mice.

	Vehicle					TAA					CTX+TAA					
Mouse number	1	2	3	4	Ave	1	2	3	4	Ave	1	2	3	4	5	Ave
HCT (%)	50.14	48.97	44.55	48.49	48.04	55.95	61.11	31.73	54.13	50.73	47.99	59.82	50.42	27.91	53.79	47.99
**HGB (g/dL)**	18.33	23.34	22.64	19.97	21.07	10.9	11.9	9.35	15.76	11.98[A]	23.51	16.06	15.36	17.23	15.61	17.55[B]
MCH (pg)	17.71	23.05	25.41	19.64	21.45	19.96	14.69	15.33	15.1	16.27	24.32	13.15	15.95	15.78	15.09	16.86
MCHC (g/dL)	36.55	47.66	50.82	41.19	44.06	42.01	30.33	29.47	29.12	32.73	48.99	26.84	30.46	30.53	29.02	33.17
MCV (fL)	48	48	50	48	48.5	48	48	52	52	50	50	49	52	52	52	51
MPV (fL)	6.41	7.8	6.53	5.6	6.59	5.56	6.09	6.54	5.88	6.02	6.06	5.72	4.97	6.15	5.55	5.69
PLT (10^9/L)	80	139	159	964	333.5	969	457	131	265	455.5	392	400	865	98	348	420.6
RBC (10^12/L)	10.35	10.12	8.91	10.17	9.89	5.46	12.62	6.1	10.44	8.66	9.67	12.21	9.63	5.4	10.34	9.45
RDW-CV (%)	13.6	14.2	15.79	13.8	14.35	13.07	13.34	13.95	14.19	13.64	15.31	14.15	14.01	14.02	14.06	14.31
WBC (10^9/L)	1.78	1.97	2.89	3.91	2.64	1.87	5.42	3.97	3.05	3.58	3.14	4.47	4.3	10.33	3.01	5.05
WBC-EOS (%)	2.6	2	1.9	8.2	3.68	6.9	12	1.5	2.1	5.63	5.8	4.7	6.1	9.3	2.3	5.64
**WBC-GRAN (%)**	13.6	6.1	6.9	20.9	11.88	20	30.3	31.8	30.6	28.18[A]	26.6	20.1	31.8	35.8	27	28.26[A]
**WBC-LYM (%)**	81.7	90.1	90.3	75.4	84.38	75	64.9	52.5	60	63.1[A]	65.4	71.3	60.9	54.8	63.9	63.26[A]
WBC-MONO (#)	0	0	0	0.1	0.03	0	0.2	0.6	0.2	0.25	0.2	0.3	0.3	0.9	0.2	0.38
WBC-MONO (%)	4.7	3.8	2.8	3.7	3.75	5	4.8	15.7	9.4	8.73	8	8.6	7.3	9.4	9.1	8.48
**ALT (U/L)**	28	30	47	17	30.5	204	251	351	345	287.75[A]	1000	1000	1000	1000	1000	1000[B]
**AST GOT (U/L)**	128	69	169	57	105.75	397	430	600	547	493.5[A]	1000	1000	1000	1000	1000	1000[B]

[A, B] *$P<0.05$* (A: Compared vehicle with TAA, B: Compared TAA with CTX+TAA)*,* Bold: significant CBC list

Table S4. Sources of somatic stem cells and their outcomes in terms of hepatic differentiation

MSC source	Coating material	Step 1	Step 2	Outcome	Reference
Human amnion stem cells	Gelatin	**PVA**, EGF, FGF2, HGF, Dexa, ITS, Nico, 5-aza, **Fasudil, CHIR**	OSM, HGF, HGF, Dexa, ITS, **Fasudil**	In vitro differentiation, In vivo transplantation	This study
Human adult liver-derived stem cells	Gelatin	FBS, HGF, FGF2, Nico, 5-aza	OSM, Dexa, ITS	In vitro differentiation, In vivo transplantation	Lee J, Choi J. et al. Cells (2020)
Human bone marrow stem cells	ECM Hydrogel, Matrigel	HGF, FGF2, Nico	OSM, Dexa, ITS	In vitro differentiation	X Wei. et al. J Biomed Master Res Part A (2018)
Human amniotic epithelial stem cells	Matrigel	FBS, EGF, FGF2, HGF, Dexa, ITS	FBS, EGF, OSM, HGF, Dexa, ITS	In vitro differentiation, In vivo transplantation	Liu, Q. et al. Stem Cell Res Ther (2018)
Human umbilical cord matrix stem cells	Type I collagen	FBS, EGF, FGF2, HGF, ITS	OSM, Dexa, ITS	In vitro differentiation, In vivo transplantation	Campard, D. et al. Gastroenterology (2008)
Human adipose tissue-derived stem cells	Type I collagen	BSA, EGF, FGF1, FGF4, HGF	OSM, Dexa	In vitro differentiation, In vivo transplantation	A, Banas. et al. Hepatology (2007)

Fig. S1

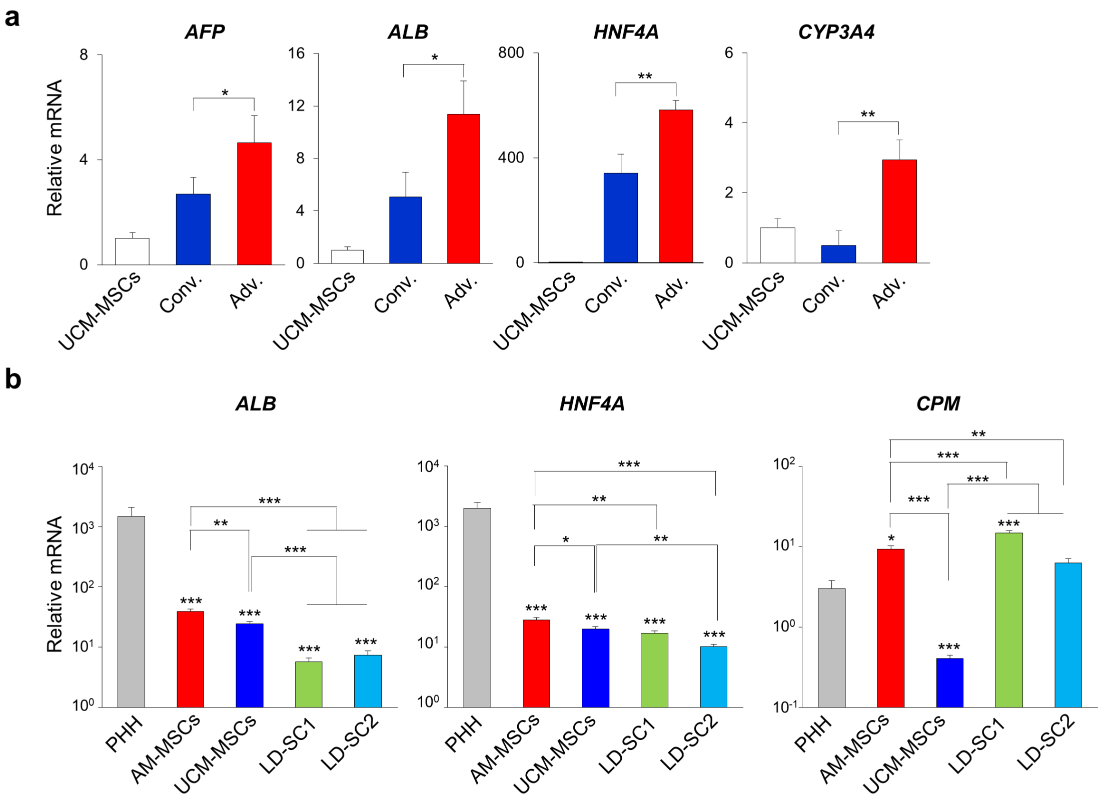
a
AFP
ALB
HNF4A
CYP3A4
Relative mRNA
UCM-MSCs
Conv.
Adv.
b
ALB
HNF4A
CPM
PHH
AM-MSCs
UCM-MSCs
LD-SC1
LD-SC2

Fig. S2

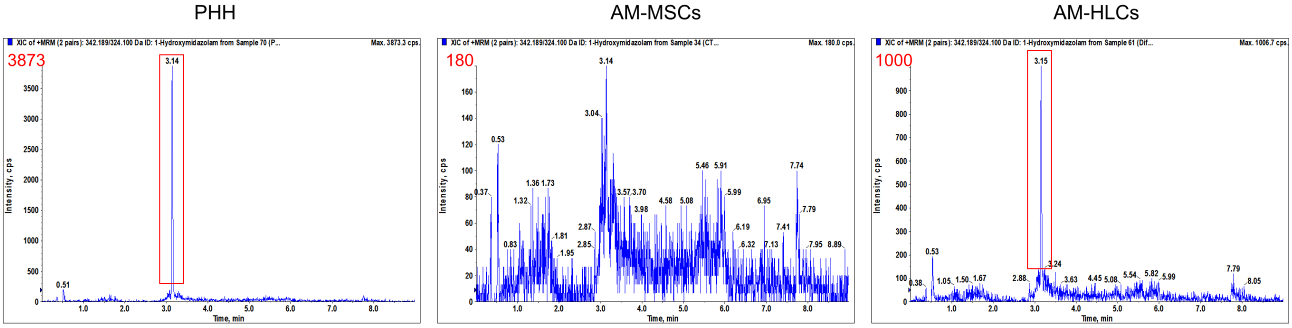
PHH
AM-MSCs
AM-HLCs
XIC of +MRM (2 pairs): 342.189/324.100 Da ID: 1-Hydroxymidazolam from Sample 70 (P...
Max. 3873.3 cps.
3873
3.14
0.51
XIC of +MRM (2 pairs): 342.189/324.100 Da ID: 1-Hydroxymidazolam from Sample 34 (CT...
Max. 180.0 cps.
180
3.14
3.04
XIC of +MRM (2 pairs): 342.189/324.100 Da ID: 1-Hydroxymidazolam from Sample 61 (Dif...
Max. 1006.7 cps.
1000
3.15
Intensity, cps
Time, min

Fig. S3

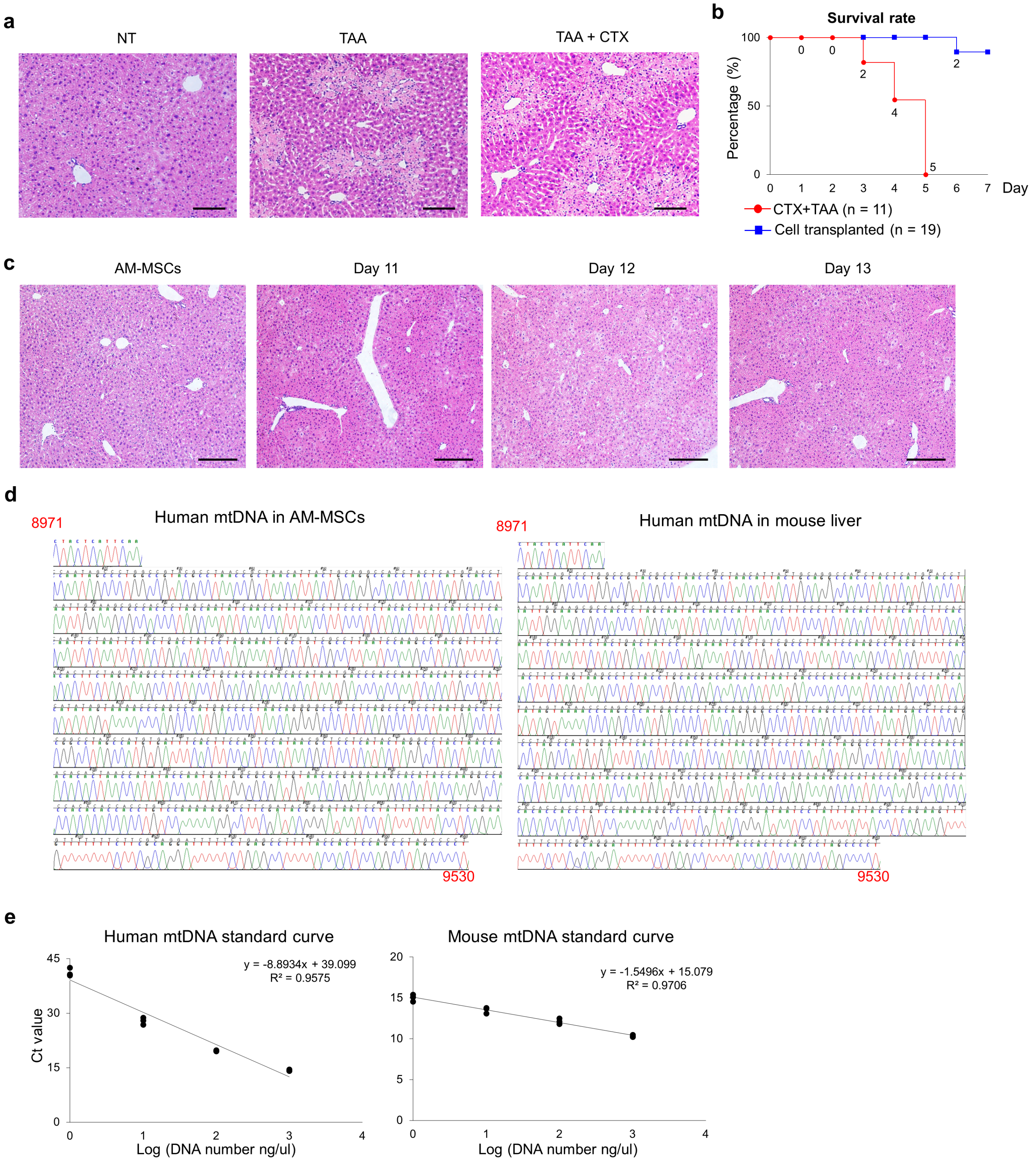
a
NT
TAA
TAA + CTX
b
Survival rate
Percentage (%)
100
50
0
0 1 2 3 4 5 6 7 Day
CTX+TAA (n = 11)
Cell transplanted (n = 19)
c
AM-MSCs
Day 11
Day 12
Day 13
d
Human mtDNA in AM-MSCs
Human mtDNA in mouse liver
8971
9530
e
Human mtDNA standard curve
y = -8.8934x + 39.099
R² = 0.9575
Ct value
Log (DNA number ng/ul)
Mouse mtDNA standard curve
y = -1.5496x + 15.079
R² = 0.9706
Log (DNA number ng/ul)
